# Structured reporting to improve transparency of analyses in prognostic marker studies

**DOI:** 10.1186/s12916-022-02304-5

**Published:** 2022-05-12

**Authors:** Willi Sauerbrei, Tim Haeussler, James Balmford, Marianne Huebner

**Affiliations:** 1 Institute for Medical Biometry and Statistics, Faculty of Medicine and Medical Center – University of Freiburg, Freiburg, Germany; 2 Department of Statistics and Probability, Michigan State University, East Lansing, MI USA

**Keywords:** Prognostic studies, Reporting guidelines, REMARK profile, Selective reporting, Bias, Transparency, Meta-analysis

## Abstract

**Background:**

Factors contributing to the lack of understanding of research studies include poor reporting practices, such as selective reporting of statistically significant findings or insufficient methodological details. Systematic reviews have shown that prognostic factor studies continue to be poorly reported, even for important aspects, such as the effective sample size. The REMARK reporting guidelines support researchers in reporting key aspects of tumor marker prognostic studies. The REMARK profile was proposed to augment these guidelines to aid in structured reporting with an emphasis on including all aspects of analyses conducted.

**Methods:**

A systematic search of prognostic factor studies was conducted, and fifteen studies published in 2015 were selected, three from each of five oncology journals. A paper was eligible for selection if it included survival outcomes and multivariable models were used in the statistical analyses. For each study, we summarized the key information in a REMARK profile consisting of details about the patient population with available variables and follow-up data, and a list of all analyses conducted.

**Results:**

Structured profiles allow an easy assessment if reporting of a study only has weaknesses or if it is poor because many relevant details are missing. Studies had incomplete reporting of exclusion of patients, missing information about the number of events, or lacked details about statistical analyses, e.g., subgroup analyses in small populations without any information about the number of events. Profiles exhibit severe weaknesses in the reporting of more than 50% of the studies. The quality of analyses was not assessed, but some profiles exhibit several deficits at a glance.

**Conclusions:**

A substantial part of prognostic factor studies is poorly reported and analyzed, with severe consequences for related systematic reviews and meta-analyses. We consider inadequate reporting of single studies as one of the most important reasons that the clinical relevance of most markers is still unclear after years of research and dozens of publications. We conclude that structured reporting is an important step to improve the quality of prognostic marker research and discuss its role in the context of selective reporting, meta-analysis, study registration, predefined statistical analysis plans, and improvement of marker research.

**Supplementary Information:**

The online version contains supplementary material available at 10.1186/s12916-022-02304-5.

## Background

As in many other fields of medicine, deficiencies in the reporting of tumor marker prognostic factor studies have long been recognized [1–3]. The Reporting Recommendations for Tumor Marker Prognostic Studies (REMARK) guidelines were developed and subsequently discussed in detail in an “explanation and elaboration” (E&E) paper [4, 5]. Prognostic factors are clinical factors used to help predict an individual patient’s risk of a future outcome, such as disease recurrence after primary treatment. Many initially promising findings of prognostic factors for cancer have failed to replicate, and very few have emerged as being clinically useful [6]. A large body of work has identified major areas of concern about the quality of much prognostic factor research, including that studies are often poorly analyzed [7] and/or selectively reported [3, 8, 9].

As highlighted in *The Lancet Reduce waste, increase value* series (e.g., [10, 11]), similar deficiencies are widespread across many fields of biomedical research. Reporting guidelines, which have been developed for a range of study designs [12], typically describe a minimum set of information that should be clearly reported, provide examples of guideline-consistent reporting, and include a checklist to facilitate compliance [13]. Adherence to reporting guidelines ensures that readers are provided with sufficient details to enable them to critically appraise a study. Good reporting also promotes greater transparency and standardization, which enhances the ability to compare and synthesize the results of different studies and thus facilitates the process of evidence synthesis and meta-analysis [14].

Unfortunately, there is convincing evidence that the publication of REMARK has not resulted in a major improvement in the quality and completeness of reporting of tumor marker prognostic factor studies [8, 14]. In a recent systematic review, Kempf et al. [9] investigated 98 prognostic factor studies published in 17 high-impact oncology journals in 2015. Almost all displayed evidence of selective reporting (i.e., the failure to present the results of all planned analyses), and most were incompletely reported (e.g., omitted essential information such as reporting a hazard ratio without its associated confidence interval). A particularly common occurrence was focusing solely on significant results in the conclusions, despite multivariable modeling revealing at least one non-significant prognostic factor effect. The presence of reporting and/or publication bias in favor of statistically significant results had already been noted over a decade ago [15].

The purpose of this paper is a structured display, “REMARK Profile,” to improve the reporting of statistical analyses conducted in tumor marker prognostic studies. This profile consists of two parts: (A) patients, treatment, and variables and (B) statistical analysis of survival outcomes. The REMARK profile is complementary to the REMARK guidelines, and a prior version was proposed and discussed in the E&E paper [5], extended with a specific example of the prognostic ability of the Nottingham Prognostic Index for breast cancer [16], and also advocated in the recent abridged version of the E&E paper written to encourage the dissemination and uptake of REMARK [17]. Our intention is to provide clear and simple examples and demonstrate how the creation of such profiles enhances the presentation and transparency of statistical analyses. The importance of transparent reporting of statistical analyses is particularly germane for observational studies (as is typical of tumor marker prognostic studies), especially where multiple exploratory analyses are included that increase the chance of spurious findings [18]. Although the REMARK guidelines focus primarily on studies of single prognostic markers, the value of a structured profile is likely to apply equally to other types of prognostic studies, including studies of multiple markers and studies of markers to predict response to treatment. Similarly, it is equally relevant to specialties other than cancer, as reflected in the fact that the REMARK guidelines have been used more widely (e.g., [19, 20]).

In this study, we produce and evaluate REMARK profiles for a selection of tumor marker prognostic studies published in five clinical journals on cancer research in 2015 (three papers from each). The paper is organized as follows. In the “Methods” section, we begin by describing the REMARK profile in greater detail, and we outline how the papers were selected and coded for analysis. In the Results section the findings are presented in two ways. First, we chose two studies which we considered to be well-reported and two studies which we considered to be less well-reported, and highlight pertinent features of each with reference to their profile. Second, we summarize and discuss the key aspects of the reporting quality of all 15 selected studies. In the Discussion section we mention several issues related to the broader role of structured reporting. We conclude that structured reporting is an important step to improve quality of prognostic marker research. A REMARK profile template is also provided with guidance to help the authors prepare profiles for their own study, ideally prospectively.

## Methods

### The REMARK profile

The REMARK profile is a structured display of relevant information designed to help authors to summarize key aspects of a tumor marker prognostic study, primarily to improve the completeness and transparency of the reporting of statistical analyses. It is intended to enable readers to quickly and accurately understand the aims of the paper, the patient population, and all statistical analyses that were carried out. The profile, if created retrospectively as in this study, can aid in assessing how well-reported a study is, identifying severe weaknesses and omissions that may call into question certain aspects of the study’s findings. Yet, ideally, if created prospectively by the authors, it might be invaluable in helping to ensure that errors and omissions do not occur in the first place. If published as Table x or as an online supplement, it could summarize relevant information without compromising the articles’ flow of reading. The profile includes much needed metadata beneficial for identifying whether a specific study fulfills inclusion and exclusion criteria for systematic reviews or meta-analyses, and the widespread use of such profiles will improve the quality and inclusiveness of primary research and reviews.

The REMARK profile consists of two sections. The first section provides information about the patient population, inclusion and exclusion criteria, the number of eligible patients and events for each outcome in the full data, how the marker of interest was handled in the analysis, and additional variables available.

The second section of the profile gives a sequential overview of all of the analyses conducted, including the variables included in each, the sample size, and the number of outcome events. It is important to also include the initial data analyses (IDA), which are a key step in the analysis workflow and aid in the correct presentation and interpretation of model results [21]. The original proposal for such a REMARK profile [5] was later extended [16] to provide more detail about the entire analysis process, including checks of important assumptions. For illustration, it is displayed in Table 1. Obviously, each study has different aspects and details of a profile differ. A simple generic profile is shown in Table 2.

**Table 1:** REMARK profile—improving the Nottingham Prognostic Index (NPI), adapted from Winzer et al. [16]

**Part a: Patients, treatments, and variables**				
**Study and marker**	**Remarks**			
Marker handled	= NPI *Continuous and categorical. Cutpoints as predefined in the literature. *For details see Blamey et al. [27] in [16]			
Further variables	v1 = Tumor size, v2 = no. of pos. Lymph nodes, v3 = tumor grade, v4 = age, v5 = histology, v6 = hormone receptor status, v7 = menopausal status, v8 = vessel invasion, v9 = lymphatic vessel invasion			
**Patients**	***n***	**Remarks**		
Assessed for eligibility	2062	**Disease:** primary breast cancer **Patient source:** Database Surgical clinic Charité, Berlin. All patients with surgery from 1^st^ Jan. 1984 to 31^st^ Dec. 1998.		
Excluded	502	63 metastasis, 73 previous carcinomas other than breast cancer, 86 primary breast cancer prior to the study, 134 breast cancer in situ, 8 pt0, 123 older than 80 years, 20 neo-adjuvant chemotherapy, 71 death within the first months of surgery, three or more standard prognostic factors missing. For some patients, more than one exclusion criterion applied		
Included	1560	Previously untreated. ***Treatment:*** *local therapy: BCT or mastectomy with or without radiotherapy, adjuvant therapy: chemo (y/n), hormone (y/n). *For details see, Add file 1 and table 2 in Winzer et al. [28] in [16]		
Outcome events	221	Overall survival: death from any cause		
**Part b: Statistical analyses**^**h**^				
**Analysis**	**Patients**	**Events**	**Variables considered**	**Results/remarks**
IDA 1a: imputation for missing values	1560	NR^b^	v1(94), v2 (68), v3(217), v6(490), v7(54)	Variables (number of patients) with imputed values
A1^c^: NPI (3)	1560	221	NPI	Prognostic value of NPI in 3 categories (Tables 2 and 3, Fig. 1)
A2: NPI (6)	1560	221	NPI	6 categories (Fig. 1, Table 3)
C1d: check of PHe in NPI (3) and in NPI (6)	1560	221	NPI	Fig. 2, S4 and non-significant result of FPT^f^ (see last paragraph 4.2)
A3: NPIcont	1560	221	NPI	More information from continuous data? (Table 3)
C2: NPIcont. has a linear effect	1560	221	NPI	FP2 function not significantly better, see 4.3.1
C3: check of PH^e^ in NPIcont	1560	221	NPI	Non-significant result of FPT^f^ (see last paragraph 4.3.1)
A4: MFP7 of the three NPI variables (univ. and multivariable)	1560	221	v1, v2, v3	Table 4
A5: functional form for nodes	1560	221	v2	Fig. 3
A6: prognostic value and additional value of further variables (univ. and multiv.)	1560	221	NPI, v4, v5, v6, v7, v8, v9	Table 5, Fig. 4
A7: MFP using all available information	1560	221	v1, v2, v3, v4, v5, v6, v7, v8, v9	Final MFP model in Table 6, see 4.5
A8: measures of separation	1560	221	NPI, v1, v2, v3, v4, v5, v6, v7, v8, v9	Table 7, see 4.6
C4: check of PH^e^ in MFP model	1560	221	v1, v2, v3, v6	Non-significant result of FPT^f^ (see end of 4.5)

^a^IDA = initial data analysis

^b^NR = not relevant

^c^A1, A2, … = number of analysis

^d^C1, C2, … = number of check

^e^PH = proportional hazards,

^f^FPT = fractional polynomial time procedure

^g^MFP = multivariable fractional polynomial procedure

^h^All analyses using a Cox model are stratified for strata according to therapy. There are 8 strata defined by the combination of surgery, radiotherapy (y/n), and systemic therapy (y/n (no chemotherapy and no hormone therapy))

**Table 2:** Generic REMARK profile

**Part a: Patients, treatments, and variables**				
**Study and marker**	**Remarks**			
Marker	M = main predictor			
Further variables	v1, v2, v3, etc.			
**Patients**	***n***	**Remarks**		
Assessed for eligibility		Disease		
		Patient source		
Excluded		Numbers and reasons for exclusions		
Included		Inclusion criteria		
Outcome(s) and number of events		Overall, perhaps also in subgroups		
**Part b: Statistical analyses**				
**Analysis**	**Patients**	**Events**	**Variables considered**	**Results/remarks**
IDA: initial data analysis	n	m	V1, V2, ...	For example, description of patient characteristics, data screening, handling of missing data
A1: univariable analysis of M			M	Page in manuscript, table, or figure
A2: model 1 or subgroup or sensitivity analysis				
C1: model evaluation or diagnostics, check of assumptions				
A3: model 2				
P1: presentation of function				

### Selected papers

Papers were selected from five clinical journals reporting on prognostic studies in cancer research. These were *Breast Cancer Research and Treatment* (BCRT), *Cancer*, *European Journal of Cancer* (EJC), *International Journal of Cancer* (IJC), and *Journal of Clinical Oncology* (JCO). *The choice of these journals was based on the earlier paper about the assessment of adherence to REMARK* [14]. *Four journals were already included in this study and here we added EJC.* A search was conducted with the search terms “cancer” in the title and “prognostic” in the title, abstract, or keywords. From each journal, three original research papers, published in 2015, were identified and reviewed, with the most recently published papers considered for eligibility first. A publication was eligible if it was a prognostic study with survival outcomes, and multivariable models were used in the statistical analysis. The exclusion criteria were randomized trials, laboratory studies, reviews, meta-analyses, methods papers, and letters. If a paper was not eligible for inclusion, the next most recent paper from that journal was selected.

The publications were summarized, including the number of patients assessed, number of patients excluded, and number of patients and events reported in the final models. Each statistical model was assessed with respect to which variables were included, number of events for the primary outcome, and whether the number of events was reported for each model or subgroup analysis. For studies that included a training and validation data set, only the training data set was considered for this summary. The studies were graded according to the completeness of information on exclusions of subjects as follows: 3, exclusion criteria and number of exclusions known; 2, exclusion criteria listed, but number of excluded patients unknown; and 1, exclusion criteria not listed.

Originally, continuous marker variables are often categorized or dichotomized for the purpose of analysis. While they technically do not represent a “new” marker, we decided to include them in the marker section of part A of the profiles for reasons of clarity and comprehensibility. An example can be seen in Martin et al. [22] with “M1” being the continuous version of the marker, and “M1(10)” or “M1(5)” describing categorized versions of the same marker with ten and five categories, respectively.

## Results

Fifteen studies from five journals were included in this review. To illustrate how REMARK profiles help readers to better understand the analysis steps in a study, we will present two positive examples in which the analyses were reported in detail and were easily understandable. Here, profiles can help readers to quickly identify that a study is well-reported and find the information needed to properly evaluate the findings. However, more frequently reporting of many important parts of the analyses is insufficient, which we will illustrate by also presenting two poorly reported studies. All fifteen profiles are available in the web appendix (Additional file 1). In the second part of the “Results” section, we will summarize our findings from them.

### Selected profiles to illustrate weaknesses of current reporting and advantages of the REMARK profile

#### Examples of better-reported studies

##### Xing et al [23]

This REMARK profile (Table 3), for a paper examining the association between BRAF V600E mutation and recurrence of papillary thyroid cancer (PTC) in eight countries between 1978 and 2011 shows at a glance that the analysis involved both univariable and multivariable analyses and employed both Cox regression (PTC regression expressed as a proportion) and Poisson regression (PTC recurrence expressed as rate per 1000 person-years). It also involves a number of subgroup analyses, including by type of PTC, and also restricting the sample to low-risk patients, defined variously as tumor stage 1, tumor stage 2, and tumor size ≤ 1.0 cm. It shows that the sample size and the effective sample size (number of events) were reported for each of these analyses. It shows that the proportional hazards assumption was checked and that a violation of this assumption resulted in the decision to stratify multivariable analyses by medical center. It shows that three nested predictive models were applied, both in analyses involving the overall sample and those restricted to subgroups: an unadjusted model including only the marker of interest (BRAF V600E mutation), a multivariable model adjusting for age and sex and stratifying by medical center, and a full model adjusting for 5 additional variables.

**Table 3:** REMARK profile for Xing et al. (2015) [23]

**Part a: Patients, treatment, and variables**					
Patients: consecutively selected patients treated for papillary thyroid cancer (PTC) at 16 medical centers in 8 countries (USA, Italy, Poland, Japan, Australia, Spain, Czech Rep, South Korea), over differing time periods spanning 1978-2011.					
**?**		Patients assessed			
**?**		Patients excluded			
**2099**		Patients included for analysis, subgroups by v8 (v8-S1: CPTC, *n* = 1448; v8-S2: FVPTC, *n* = 431), v9 (v9-S1: stage I, *n* = 1273; v9-S2: stage II, *n* = 234), and v4 (v4-S1: tumor size ≤ 1.0 cm, *n* = 534) Missing data not mentioned—appears to have been none			
Treatment and follow-up: total thyroidectomy and neck dissection in all patients, postoperative hormone suppression, and radioiodine ablation (in all centers except the Japanese center). Median follow-up 36 months (IQR 14 to 75 months)					
Marker:		M = BRAF V600E mutation (positive/negative)			
Outcome (events)		Recurrence free survival (RFS, events overall: *n* = 338; in subgroups: v8-S1: *n* = 247, v8-S2: *n* = 43, v9-S1: *n* = 119, v9-S2: *n* = 32, v4-S1: *n* = 57). Expressed as both a proportion of recurrences, and as rate of recurrence per 1000 person-years of follow-up			
Further variables		v1 = age, v2 = sex, v3 = medical center, v4 = tumor size, v5 = extrathyroidal invasion, v6 = lymph node metastasis, v7 = multifocality, v8 = PTC subtype, v9 = tumor stage Adjustment model 2: v1–v3; model 3: v1–v8			
**Part b: Statistical analysis of survival outcomes**					
Aim		*n*	Outcome (events)	Variables considered	Results/remarks
Ch1: check of proportional hazards assumption after initially fitting models A2 and A3					Led to stratification by medical center (v3) and revision of these analyses
IDA1: computation of rates of recurrence per 1000 person-years		Total and all subgroups			Displayed in Tables 2 and 4 and A3
A1: univariable unadjusted model 1	All	2099	RFS (338)	M	Poisson regression *p*-values and CI; Cox regression HR, CI: Table 2; Kaplan-Meier estimates of recurrence-free survival, *p*-values: Fig. 1
	v8-S1	1448	RFS (247)		
	v8-S2	431	RFS (43)		
A2: multivariable model 2	All	2099	RFS (338)	M, v1–v3	*p*-values, HR, CI, Table 2
	v8-S1	1448	RFS (247)		
	v8-S2	431	RFS (43)		
A3: multivariable model 3	All	2099	RFS (338)	M, v1–v8	*p*-values, HR, CI, Table 2
	v8-S1	1448	RFS (247)		
	v8-S2	431	RFS (43)		
A4: sensitivity analysis, excluding patients with < 3 year follow-up, no recurrence		?	RFS ?	M	Results p.44 text. Data not shown
A5: interaction of M with conventional risk factors, univariable		2099	RFS (338)	M, v1 (dichotomized), v5, v6	Kaplan-Meier estimates, *p*-values, Fig. 2, Synergy Index, CI, Table 3
A6: low-risk patients, unadjusted model 1	v9-S1	1273	RFS (119)	M	Poisson regression *p*-values and CI; Cox regression HR, CI: Table 4
	v9-S2	234	RFS (32)		
	v4-S1	534	RFS (57)		
A7: low-risk patients, multivariable model 2	v9-S1	1273	RFS (119)	M, v1–v3	*p*-values, HR, CI, Table 4
	v9-S2	234	RFS (32)		
	v4-S1	534	RFS (57)		
A8: low-risk patients, multivariable model 3	v9-S1	1273	RFS (119)	M, v1–v8	*p*-values, HR, CI, Table 4
	v9-S2	234	RFS (32)		
	v4-S1	534	RFS (57)		
A9: univariable model in v4 subgroups		Varies by subgroup	RFS (varies)	M	*p*-values, HR, CI, Tab. A2
A10: univariable model for 35 subgroups by v1, v2, and v8		Varies by subgroup	RFS (Varies)	M	HR, CI, Tab. A4

Statistical software packages used: SAS v.9.3

*CPTC* conventional PTC, *FVPTC* follicular-variant PTC

The profile also reveals two minor reporting deficiencies. The number of patients assessed for eligibility is not provided, nor is the number of exclusions (or indeed whether there were any exclusion criteria). There is also no mention of missing data, though it appears that there may have been none.

##### Huzell et al [24]

This profile (Table 4) summarizes a paper exploring the effect of oral contraceptive use on breast cancer events and distant metastasis among Swedish patients diagnosed with primary breast cancer between 2002 and 2011 and followed up for a median of 3 years. The analyses are complex, with the marker categorized in 5 different ways and a number of subgroups explored. In general, however, the profile shows that the reporting of key information is quite good, with the *n*’s and number of outcome events for each analysis known (with the exception of the subgroup analyses in which distant metastasis was the outcome) and clear statements on missing data in Tables 1 and 2 of Ref. [22]. The profile is particularly valuable as many analyses were conducted and some were only briefly mentioned in the text of the results section. For some analyses (e.g., A1 and A4), no data are provided. Thus, the profile greatly helps to clarify what was done, including to clarify which covariates were included in each analysis.

**Table 4:** REMARK profile for Huzell et al. (2015) [24]

**Part a: Patients, treatment, and variables**					
Patients: diagnosis of primary breast cancer 2002–2011 at Skåne University Hospital Lund, Sweden					
**1045** **51** **994**	Patients assessed Patients excluded (treatment prior to surgery) Patients included for descriptive analysis				
**46**	Patients additionally excluded (in situ carcinoma, 38; metastatic spread within 3 months, 8)				
**948**	Patients included for predictive analysis of the risk of an early breast cancer (BC) event				
Treatment and follow-up: standard care. Follow-up up to 9 years (until December 2012); median 3.03 years (IQR 1.93–5.23)					
Markers	Oral contraceptive (OC) use: M1 = ever OC use (yes/no) M2 = se before age 20 (yes/no) M3 = OC use before first child (yes/no) M4 = OC start 1974 or later (proxy for dose) (yes/no) M5 = duration of OC use (continuous)				
Outcomes (events)	Breast cancer events (BCE) (100): distant metastasis (DM) (65)				
Variables	v1 = age, v1_c50 = age ≥ 50 (proxy for menopause), v2 = tumor size^a^, v3 = grade^b^, v4 = nodal involvement, v5 = hormone receptor status, v6 = BMI^9^, v7 = endocrine treatment				
Missing data	See Tables 1 and 2				
**Part b: Statistical analysis of survival outcomes**					
Aim	*n*	Events	Outcome	Variables considered	Results/remarks
IDA1: data screening and definitions of categories	994	NA		M1, M2, M3	Stat. methods. Definition of markers (categorization of OC use)
IDA2: descriptive	994	NA	OC use categories (M1, M2, M3)	v1–6, plus 12 descriptive-only variables	Table 1 (patient characteristics), Table 2 (tumor characteristics)
A1: multivariable	948	100	BCE	M1, M2, M3, v1-v6	Reported only in the text (no data provided), p.508 first column
A2: univariable/multivariable subgroup v1_c50 ≥ 50	760	70	BCE	M1, M2, M3, v1–v6	For M2: Fig. 2a, Table 3. Kaplan-Meier, log-rank, and HR. M1 and M3 non-significant and reported only in the text, p.508 first column
A3: univariable/multivariable subgroup v1_c50 < 50	188	30	BCE	M1, M2, M3, v1–v6	For M2: Fig. 2b, Table 3. Kaplan-Meier, log-rank, and HR. M1 and M3 non-significant and reported only in the text, p.508 first column
A4: multivariable subgroup v1_c50 ≥ 50	?	?	DM	M2; v1–v6	Reported in the text, no data provided: p.508 second column
A5: multivariable subgroup v1_c50 < 50	?	?	DM	M2; v1–v6	Log-rank and HR in text, p.508 second column
A6: multivariable	948	100	BCE	M4; v1–v6	HR in text, p.508 second column
A7: multivariable subgroup v1_c50 ≥ 50	760	70	BCE	M4, M5; v1–v6	HR in text, p.508 second column
A8: multivariable subgroup v1_c50 < 50	188	30	BCE	M4, M5; v1–v6	HR in text, p.508 second column
A9: multivariable subgroup v7 (TAM treatment), v1_c50 (age ≥ 50), v5 (ER+)	372	29	BCE	M1; v1–v7	Fig. 3a. Kaplan-Meier, log-rank, and HR—adjusted for tumor and patient characteristics and aromatase inhibitor (AI) treatment
A10: multivariable subgroup v7 (AI treatment), v1_c50 (age ≥ 50), v5 (ER+)	277	26	BCE	M1; v1–v7	Fig. 3b. Kaplan-Meier, log-rank, and HR—adjusted for tumor and patient characteristics and tamoxifen (TAM) treatment

Statistical software packages used: SPSS v.19

*BMI* body mass index, *BCE* breast cancer event = local or regional recurrence, distant metastasis, or contralateral breast cancer

^a^Invasive tumor size ≥ 21 or muscle or skin involvement (yes/no) in the multivariable model

^b^Grades I–II vs grade III in the multivariable model

9BMI ≥ 25 kg/m2 (yes/no) in the multivariable model

#### Examples of inadequately reported studies

##### Thurner et al. [25]

This profile (Table 5) summarizes an analysis of the effect of pre-treatment C-reactive protein on three clinical outcomes (cancer-specific survival, overall survival, and disease-free survival) in prostate cancer patients. All received 3D radiation therapy and were followed up for a median of 80 months. Five clinical variables are included in models as potential covariates, while a sixth (risk group) is used in subgroup analyses. The numbers of patients both initially assessed and subsequently excluded are not provided, as is clear from the profile.

**Table 5:** REMARK profile for Thurner et al. (2015) [25]

**Part a: Patients, treatment, and variables**				
Patients: treated for primary prostate cancer at the Department of Therapeutic Radiology and Oncology, Medical University of Graz, Austria, 2003–2007				
**> 700**	Patients assessed			
**> 439**	Patients excluded (did not meet below criteria, as well as those with a follow-up of < 4 months)^a^			
**261**	Patients included for analysis (histologically confirmed primary prostate cancer + pre-treatment CRP levels taken)			
Treatment and follow-up: 3D radiation therapy in curative intent; median follow-up 80 months				
Markers	M = pre-treatment CRP (continuous variable; analyses for dichotomized or categorical data, based on optimal cutpoints)			
Outcomes (events)	CSS—primary outcome (24), OS (59), DFS (56)			
Further variables	v1 = age at diagnosis, v2 = PSA at diagnosis, v3 = tumor stage, v4 = Gleason score, v5 = risk group^b^, v6 = total duration of ADT			
**Part b: Statistical analysis of survival outcomes**				
Aim	*n*	Outcome (events)	Variables considered	Results/remarks
IDA1: correlations	Varies^c^		M, v1–v5	Results p.613 first column
IDA2: determination of optimal cutpoint for M	261	CSS (24)	M	CRP dichotomised into high (≥ 8.6 mg 1^−1^) and low (< 8.6 mg ^-−1^)
A1: univariable survival analysis	261	A1.1 CSS (24) A1.2 OS (59) A1.3 DFS (56)	M	Kaplan-Meier estimates, Figs. 1, 2, and 3
A2: univariable associations	Varies	A2.1 CSS (24) A2.2 OS (59) A2.3 DFS (56)	M, v1, v2, v3, v4, v6	HR, CI, *p*-values, Tables 2, 3, and 4
A3: multivariable (including v. from A2.1, A2.2, and A2.3 with *p*<.05)	?	CSS (?) OS (?) DFS (?)	M, v2, v3, v4, v6	HR, CI, *p*-values, Table 2 (CSS), Table 3 (OS), Table 4 (DFS)
A4: univariable, high risk (v5)	144	A4.1 CSS (?) A4.2 OS (?) A4.3 DFS (?)	M	HR, CI, *p*-value, Table 5
A5: multivariable, high risk (v5)	144	A5.1 CSS (?) A5.2 DFS (?)	M, v6	HR, CI, *p*-value, Table 5^d^
A6: univariable, intermediate risk (v5)	66	A6.1 CSS (?) A6.2 OS (?) A6.3 DFS (?)	M	*p*-values in text, p.615 first column (all n.s.)
A7: univariable, low risk (v5)	51	A7.1 CSS (?) A7.2 OS (?) A7.3 DFS (?)	M	*p* -values in text, p.615 first column (all n.s.)
IDA3: cutpoint determination for M in subgroups of v5	261	CSS (24)	M	CRP categorized with cutoff values of 8.9, 8.4, and 13.4 for the v5 risk groups
A8: univariable by v5 subgroups	144/66/51	A8.1 high-risk, CSS (?) A8.2 intermediate-risk, CSS (?) A8.3 low-risk, CSS (?)	M	No data shown, p.615 first column (findings same as A4.1, A6.1, and A7.1)
IDA4: cutpoint determination for M in subgroups of v6^e^	261	CSS (24)	M	CRP dichotomized with cutoff values of 6.7 and 8.9 for patients with and without ADT
A9: univariable by v6 subgroups	?/?	A9.1 CSS (?) A9.2 OS (?) A9.3 DFS (?)	M	HR, CI, *p*-value, p.615 first and second columns

Statistical software packages used: SPSS v.20

*CRP* C-reactive protein, *CSS* cancer-specific survival: time from diagnosis to date of prostate cancer-related death, *OS* overall survival, *DFS* clinical disease-free survival, *ADT* androgen deprivation therapy

^a^It is not stated how many patients had a follow-up of < 4 months, nor whether these were excluded prior to the final 261 or were excluded from the 261 in subsequent analyses. We will assume the former

^b^Three categories

^c^Due to missing data. Numbers are available in Table 1

^d^No multivariable analysis was carried out for OS because v6 was not significant at A4

^e^Here defined as with/without ADT

The marker variable (C-reactive protein) is initially dichotomized on the basis of a ROC curve analysis (no details given), and a series of univariable and multivariable models are applied to the full data set. Dichotomization, although known to have severe weaknesses [7], is used in the overall population and in subgroups (IDA2, IDA3). Unsurprisingly, different cutpoints were identified in different populations. While the amount of missing data for individual variables is provided, the number of patients included in multivariable models including combinations of these variables is not provided, and consequently, the number of outcome events for these analyses is not known. In subgroup analyses by risk group, the number of outcome events is never provided. Overall, the profile effectively communicates the complexity of the analyses, much of the detail of which is hidden in the text of the results section rather than reported in any tables (see the remarks for A6, A7, and A8), and the omission of important data on the number of outcome events in all subgroup analyses.

##### Schirripa et al. [26]

This study evaluated the role of NRAS mutations as a prognostic marker in metastatic colorectal cancer (mCRC), among 786 patients treated at the University Hospital of Pisa from 2009 to 2012. Patients were categorized as having a NRAS mutation, KRAS mutation, BRAF mutation, or none of the above (all wild type). The primary outcome was overall survival, without any information about follow-up time. A number of demographic and clinical variables were examined for their relation to overall survival, some of which were selected for inclusion in multivariable models. These survival models compared the three types of mutation with the wild-type category.

The REMARK profile prepared for this paper (Table 6) reveals a number of important omissions and questionable practices. As well as the failure to specify the follow-up period, the number of events was unspecified for the overall survival. It is also unstated whether all patients with mCRC with available data and treated in the specified time period were included in the analysis, or whether there were other exclusion criteria. There were missing data for some of the covariates (see Table 1 of Ref. [26]), and as a result, an unstated number of observations are excluded in each of the multivariable models presented; that is, for each model, both the number of observations and the number of outcome events are unknown.

**Table 6:** REMARK profile for Schirripa et al. (2014) [26]

**Part a: Patients, treatment, and variables**				
Patients: tissue samples from patients with metastatic colorectal cancer (mCRC) from 2009 to 2012 were analyzed at the Pathology Department of the University Hospital of Pisa				
**?**	Patients with available KRAS, BRAF, and NRAS mutational status included			
**?**	Patients excluded			
**786**	Patients included for analysis			
Treatment and follow-up: follow-up not mentioned				
Markers	M1 = NRAS mutation (y/n), M2 = KRAS mutation (y/n), M3 = BRAF mutation (y/n), M4 = all wt (no NRAS, KRAS or BRAF mutation) (y/n)^a^			
Outcomes (events)	OS (?), PFS (?)			
Further variables	v1 = sex, v2 = age at diagnosis, v3 = ECOG PS (0/1–2), v4 = primary tumor site (nominal), v5 = mucinous histology (y/n), v6 = tumoral penetration (pT) (1–2/3–4), v7 = nodal involvement (pN) (0/1–2), v8 = time to metastasis (mts) (binary), v9 = number of mts (1/> 1), v10 = resected primary (y/n), v11 = liver only mts (y/n), v12 = liver mts (y/n), v13 = lung mts (y/n), v14 = nodes mts (y/n), v15 = peritoneal mts (y/n), v16 = bone mts (y/n), v17 = metastasis site (v11–v16) classified into 6 categories; see Table 2			
**Part b: Statistical analysis of survival outcomes**				
Aim	*n*	Outcome (events)	Variables considered	Results/remarks
IDA: homogeneity	786 various n due to missing	–	M1–M4, v1–v9, v11–v17	*p*-values, Tables 1 and 2
A1: univariable	786	OS (?)	M1- M4	Kaplan-Meier-estimate, Log-rank-test (p-value) Fig. 1
A2: univariable	321 (47 (M1) + 274 (M4), see Table 1)	OS (?)	M1, M4	Kaplan-Meier estimate, HR, CI, *p*-value, Fig. 2
A3: univariable	Varies	OS (?)	M1–M4, v3–v5, v8, v10, v11	HR, CI, *p*-value, Table 3^b^
A4: multivariable M1 vs M4, M2 vs M4, and M3 vs M4	Varies but unknown	OS (?)	Adjusted for v3–v5, v8, v10, v11	HR, CI, *p*-value, Table 4
Additional: NRAS patients treated with anti-EGFR monoclonal antibodies	8	Median OS and PFS		See page 87

Statistical software packages used: no information given

*OS* overall survival (time from diagnosis of metastatic disease to death of al causes), *PFS* progression-free survival (time from the beginning of treatment to disease progression or death of any cause)

^a^Tested for NRAS mutation only in patients with wtKRAS and wtBRAF

^b^Only significant analyses are shown in Table 3. What about others, e.g., v7: non-significant? No statement

The paper is also an example of two problems which are widespread in the literature. The first is only reporting univariable analyses which were statistically significant and omitting information about the other variables investigated. For example, it cannot be ascertained whether variable v7 (nodal involvement) was not investigated, or whether it was simply non-significant. The second problem is the use of the results of univariable analyses to select variables for inclusion in multivariable models, which is not recommended, mainly because it can lead to the exclusion of important covariates [27]. Finally, the statistical software used to carry out the analyses is not specified.

#### Summary of the quality of reporting

While the final number of patients included in the analyses was consistently reported (though incorrectly in one publication), complete information on how many patients were assessed or excluded was missing in 67% (10 of 15) of the publications (Table 7). Four studies (27%) did not provide the time period over which patients were selected for inclusion.

**Table 7:** The 15 publications with number of patients and follow-up information

ID	Study	Journal	Country/year	Data source	Number of patients			Follow-up
					Assessed	Excluded	Included	
***b1***	Hayashi (Hayashi et al. 2015) [28]	BCRT	Japan/2001–2012	Multiple institutional databases	1466	1034	432	Median 50.6 months
***b2***	Huzell (Huzell et al. 2015) [24]	BCRT	Sweden/2002–2011	Cohort	1045	97	948	Median 3 years
***b3***	Jerzak (Jerzak et al. 2015) [29]	BCRT	Canada/2007	Institutional database	Unknown	Unknown	129	Min 5 years
***c1***	Billingsley (Billingsley et al. 2015) [30]	Cancer	USA/years unknown	Cohort	544	9	535	Median 68 months
***c2***	Huang (Huang et al. 2015) [31]	Cancer	Canada/2000–2010	Cohort	1108	406	702	Median 5.1 years
***c3***	Price (Price et al. 2015) [32]	Cancer	Australia/2006–?	Registry	Unknown	Unknown	2972	Not reported
***e1***	Gonzalez-Vallinas^a^ (González-Vallinas et al. 2015) [33]	EJC	Spain/2000–2004	Institutional database	Unknown	Unknown	77	Median 72 months
***e2***	Hokuto (Hokuto et al. 2015) [34]	EJC	Japan/2000–2012	Institutional database	Unknown	Unknown	150	Median 51.8 months
***e3***	Thurner (Thurner et al. 2015) [25]	EJC	Austria/2003–2007	Institutional database	> 700	> 439	261	Median 80 months
***i1***	Keck (Keck et al. 2015) [35]	IJC	Germany/1982–2007	Institutional database	473 (?)	226 (?)	247	Up to 15 years
***i2***	Roedel (Rödel et al. 2015) [36]	IJC	Germany/years unknown	Multiple institutional databases	Unknown	Unknown	95	Median 40 months, range 1–264
***i3***	Schirripa (Schirripa et al. 2015) [26]	IJC	Italy/2009-2012	Institutional database	Unknown	Unknown	786	Not reported
***j1***	Martin^a^ (Martin et al. 2015) [22]	JCO	Multiple countries/years unknown	Cohorts	8737	577	8160	Median 41.3 months
***j2***	Ostronoff^a^ (Ostronoff et al. 2015) [37]	JCO	UK/1992–2009	Clinical trial data sets	Unknown	Unknown	156	Not reported
***j3***	Xing (Xing et al. 2015) [23]	JCO	Multiple countries/1978–2011	Cohorts	Unknown	Unknown	2099	Median 36 months, quartiles (14,75)

*BCRT* Breast Cancer Research and Treatment, *EJC* European Journal of Cancer, *IJC* International Journal of Cancer *JCO* Journal of Clinical Oncology

^a^Study with training/validation data sets: only training sample considered for this table

The number of events for the primary outcome among the total number of included patients was missing in 40% (6 of 15) of the publications (Table 8). More frequently, however, the number of events for multivariable models could not be ascertained because of missing data for one or more covariates. While for such models the number of observations was generally reported, it was often not known whether the exclusions were event cases or non-events. Of the 9 publications which reported the total number of events, five [22, 25, 28–30] were affected by this problem.

**Table 8:** Overview of several criteria and assessment of the quality of reporting

ID	Study	Journal	Markers	Outcomes	Variables	Events for primary outcome	Events for all outcomes reported	Information on exclusions^**a**^	Subgroup analysis^**b**^
**b1**	Hayashi	BCRT	1	1	3	Unknown	No	3^c^	
**b2**	Huzell	BCRT	1	2	7	100	Yes	3	2
**b3**	Jerzak	BCRT	2	2	14	36	Yes	2	
**c1**	Billingsley	Cancer	1	2	8	Unknown	No	3	
**c2**	Huang	Cancer	3	2	8	257	Yes	3	2
**c3**	Price	Cancer	1	1	9	Unknown	No	1	0
**e1**	Gonzalez-Vallinas	EJC	1	1	9	22	Yes	2	
**e2**	Hokuto	EJC	1	5	13	86	No	1	
**e3**	Thurner	EJC	1	3	6	24	Yes	2	0
**i1**	Keck	IJC	2	2	10	Unknown	No	1	0
**i2**	Roedel	IJC	2	4	7	27	Yes	1	1
**i3**	Schirripa	IJC	3	1	11	Unknown	No	1	0
**j1**	Martin	JCO	2	1	8	6294	Yes	3	2
**j2**	Ostronoff	JCO	2	6	10	Unknown	No	2	2
**j3**	Xing	JCO	1	1	9	338	Yes	1	2

*BCRT* Breast Cancer Research and Treatment, *EJC* European Journal of Cancer, *IJC* International Journal of Cancer, *JCO* Journal of Clinical Oncology

^a^Completeness of information on exclusions: 3, exclusion criteria and number of exclusions known; 2, exclusion criteria listed, but number of excluded patients unknown; and 1, exclusion criteria not listed

^b^Subgroup analysis: 2, subgroup analyses performed and sample size and number of events given for at least one subgroup analysis; 1, subgroup analyses performed and sample size given for at least one subgroup analysis, but number of events not known; and 0, subgroup analyses performed, but no sample size or number of events given

^c^Note: reference to a previous study by the authors is required

Follow-up was commonly reported as the median follow-up, while some authors included minimum, maximum, or range of follow-up. In 3 publications (20%), the duration of follow-up was not reported.

Sample sizes and number of events were often missing for subgroup analyses. Of the 10 studies with subgroup analyses, only 5 stated both the sample size and the number of events for at least one of the subgroup analyses. A further publication provided the sample size, but the number of events was not reported.

The type and version of the statistical software used in the analysis were mentioned in 10 of the 15 papers.

## Discussion

Nearly forty years ago Altman et al. [38] proposed statistical guidelines for the contributor to medical journals; about a decade later, Lang and Secic [39] published a book about how to report statistics in medicine, and Lang and Altman [40] published the SAMPL (Statistical Analyses and Methods in the Published Literature) guidelines. They state “The truth is that the problem of poor statistical reporting is long-standing, widespread, potentially serious, concerns mostly basic statistics and yet is largely unsuspected by most readers of the biomedical literature,” and in a study assessing reporting quality of about 400 research papers, Diong et al. [41] conclude that there is no evidence that reporting practices improved following the publication of editorial advice. Obviously, severe improvement is urgently needed. Suitable ideas, such as tables to replace text [42] and a list of key points giving guidance for conducting confirmatory prognostic factor studies [43], can be helpful.

Reporting guidelines have been published and it has been proposed to summarize key issues of a study, including all steps of the analysis, in a REMARK profile [4, 5, 17]. Our review of 15 prognostic factor studies demonstrated poor reporting of analyses, with relevant information, such as years of patient selection, number of patients assessed, years of follow-up, and number of events, missing. Furthermore, if available, this information may not have been clearly presented or easy to find in the paper. REMARK profiles augment the more detailed REMARK guidelines and empower researchers to prospectively report sequential analyses to provide sufficient information in a brief and clear structure. We present several reasons why this format should be adopted by researchers.

### Structured profiles to improve reporting bias and related consequences for meta-analyses

Weaknesses of analyses have been known for a long time from seminal papers about statistical aspects and methodological challenges of prognostic factor studies [44, 45]. With an emphasis on all statistical analyses conducted, we summarized the information according to the principles of the REMARK profile [5] and some extensions [16]. In a book providing a broad overview and summarizing the major reporting guidelines in health research, Altman et al. stressed the importance of structured reporting and selected the REMARK profile as one of their *creators’ preferred bits* [46, 47]. Two reviews of prognostic factor studies showed that adherence to the REMARK reporting guidelines is lacking [14, 48], but according to our knowledge, this is the first study that provides structured profiles for a group of systematically selected study publications. Unfortunately, we must assume that most of the studies lacked a prospective statistical analysis plan (SAP), and it is likely that many more analyses were conducted in many studies and that the reporting bias is therefore strong.

It is well-known that problems from the design, analysis, and reporting from single studies cause severe problems for subsequent systematic reviews and meta-analyses, specifically in the context of observational studies. Already 20 years ago, Doug Altman [49] stated *As a consequence of the poor quality of research, prognostic markers may remain under investigation for many years after initial studies without any resolution of the uncertainty. Multiple separate and uncoordinated studies may actually delay the process of defining the role of prognostic markers*. Subsequent research and empirical evaluations have shown his concerns were justified. In a large systematic review of tumor markers for neuroblastoma, Riley et al. [1] identified 130 different markers in 260 studies. They identified severe problems in both statistical analysis and presentation which restricted both the extraction of data and the meta-analysis of results from the primary studies. In a paper entitled *Prognostic Factors – confusion caused by bad quality of design, analysis and reporting of many studies*, Sauerbrei [50] discussed several critical issues in data analysis and summary assessment of a prognostic factor. It is well accepted that the concept of evidence-based medicine is a key part of research and decision-making for the assessment and comparison of treatments. As EBM requires suitable systematic reviews and meta-analyses, it is still a long way until this concept becomes reality for the use of prognostic markers in patient handling [51].

This unfortunate situation is also well known to many clinicians and it is frustrating to witness that several markers are investigated for a long time without being able to assess their clinical utility. Malats et al. [52] reviewed 168 publications from 117 studies assessing the value of P53 as a prognostic marker for bladder cancer. They conclude *After 10 years of research, evidence is not sufficient to conclude whether changes in P53 act as markers of outcome in patients with bladder cancer*’ and state *That a decade of research on P53 and bladder cancer has not placed us in a better position to draw conclusions relevant to the clinical management of patients is frustrating*.

The cited papers were published at the beginning of the century and REMARK guidelines, which were published in 2005, were still unknown to the authors. Since then, there have been many important proposals to improve prognostic marker research (see below), but it is still not uncommon that systematic reviews and meta-analyses of prognostic markers have severe weaknesses and do not provide evidence-supported knowledge about the clinical value of a marker. In a systematic review, Papadakis et al. [53] identified 20 studies investigating BAG-1 as a marker in early breast cancer prognosis. They assessed the quality of reporting according to the REMARK guidelines and conducted three meta-analyses. Sauerbrei and Haeussler [54] criticized several major weaknesses in the quality of reporting and meta-analyses and concluded that results and inferences from the study were not justified by the assessments and analyses presented. An inadequate assessment of the quality of reporting according to REMARK is the first issue they mention.

### Only a small number of markers accepted and used in practice

It is often critiqued that only a small number of markers is generally accepted and used in practice [2]. Weaknesses of bad reporting of single studies are among the main reasons for this unfortunate situation. Bad reporting causes severe problems to conduct a systematic review followed by an informative meta-analysis, which aims to provide an unbiased estimate of the effect of a variable. Many markers could not show their value in a meta-analysis, and we should be pleased that they are hardly accepted and used in practice.

Kyzas et al. [3] published a meta-analysis of the tumor suppressor protein TP53 as a prognostic factor in head and neck cancer. The authors provide compelling empirical evidence that selective reporting biases are a major impediment to conducting meaningful meta-analyses of prognostic marker studies. In a related editorial, McShane et al. [2] discuss that these biases have serious implications, not only for meta-analyses but also for the interpretation of the cancer prognostic literature as a whole. They summarize *The number of cancer prognostic markers that have been validated as clinically useful is pitifully small …*, and 2 years later Real and Malats [55] state *The saga of replication failures in prognostic-marker studies is frustrating: no new molecular markers have yet been incorporated into clinical practice for bladder cancer*. The messages from educational and methodological papers were very clear, but publishing reporting guidelines was not sufficient to help improve this unfortunate situation. Seven years after the publication of the REMARK guidelines, Kern [56] states in a paper entitled *Why your new cancer biomarker may never work: recurrent patterns and remarkable diversity in biomarker failures* that less than 1% of published cancer biomarkers actually enter clinical practice. He also discusses systematic attempts to improve marker development and adoption but *who’s listening*, a question asked in the more general context of reducing waste in biomedical research [57].

### Guidelines for different study designs and the consequences of insufficient reporting

The development of reporting guidelines started with CONSORT for randomized trials [58], which were updated several times. The CONSORT statement is required in many journals and has led to more clarity and details in the reporting of such studies. It provides more background to readers to appropriately evaluate the significance of the studies and helps to better assess the reported results. Realizing the advantages further guidelines were developed for many types of observational studies [59, 60], with the EQUATOR network [61] serving as a coordinating center [12]. Meanwhile, hundreds of reporting guidelines have been developed. To improve and partly standardize this process, Moher et al. [62] proposed guidance for developing a reporting guideline in health research.

For the reporting of systematic reviews, the Preferred Reporting Items for Systematic Reviews and Meta-Analyses (PRISMA) statement was published, with an updated version (the PRISMA 2020 statement) recently [63]. Systematic reviews and meta-analyses are the key parts of evidence-based medicine and consequently also for decision-making in patient handling, clearly illustrating the importance of the guideline for practice.

To extend REMARK to a reporting guideline for multivariable prediction models, where several prognostic covariates are combined to make individualized predictions, the Transparent Reporting of a multivariable prediction model for Individual Prognosis Or Diagnosis (TRIPOD) initiative published the TRIPOD statement with a corresponding explanation and elaboration paper [64, 65]. To assess the completeness of reporting of prediction model studies published just before the introduction of the TRIPOD statement, Heus et al. [66] conducted a review in journals with high impact factors. They found that more than half of the items considered essential for transparent reporting were not fully addressed in publications and that essential information for using a model in individual risk prediction, i.e., model specifications and model performance, was incomplete for more than 80% of the models. For (nearly) all common diseases, many prediction models and sometimes even related tools are developed, but most of them are never used in practice [67, 68]. A quarter of a century ago, Wyatt and Altman [69] published a commentary entitled *Prognostic models: Clinically useful or quickly forgotten?* The empirical evidence of poor reporting provides one of the explanations that many prediction models cannot be used in practice and are quickly forgotten.

For a systematic review of prediction models, the Checklist for critical Appraisal and data extraction for systematic Reviews of prediction Modelling Studies (CHARMS) was developed [70]. These guidelines were used to assess the methodological quality of prognostic models applied to resectable pancreatic cancer [71]. The authors provide evidence of severe weaknesses, and for improvement in the future, they highlight issues relating to general aspects of model development and reporting, applicability of models, and sources of bias. Due to a lack of standardization of reporting of outcomes, a meta-analysis could not be performed.

Consequences of bad reporting and the severity of problems it causes in the assessment of prediction models for COVID-19 were recently illustrated. Wynants et al. [72] conducted a systematic review and critical appraisal (up to 5 May 2020) of prediction models for diagnosis and prognosis. Their summary is shattering “*…proposed models are poorly reported, at high risk of bias, and their reported performance is probably optimistic. Hence, we do not recommend any of these reported prediction models for use in current practice.*” This sentiment is echoed in an Editorial by Sperrin et al. [73] who argue that the urgency of the situation cannot excuse “*methodological shortcuts and poor adherence to guidelines*,” as hastily developed models might “*do more harm than good*.”

REMARK and TRIPOD were developed for markers and models based on clinical data, having not more than some dozens of potential predictors in mind. Obviously, problems of analysis and reporting are more severe in high-dimensional data, which provide many new opportunities for clinical research and patient handling. In order to extract the relevant information from such complex data sets, machine learning, artificial intelligence, and more complicated statistical methods are often used to analyze the data. Obviously, it is important that techniques used adhere to established methodological standards already defined in prognostic factor and prediction model research [74]. Concerning patients’ benefit from the use of machine learning and artificial intelligence techniques, Vollmer et al. [75] ask *20 critical questions on transparency, replicability, ethics, and effectiveness*. To present machine learning model information, a *model facts* label was recently proposed [76]. If adopted widely, it can become an important instrument to severely improve the clinical usefulness of machine learning models.

Including in the supplemental information, a reproducible report (Markdown or Jupyter Notebook) with all the code for the statistical analyses would be another suitable way to report analyses of gene expression data and all associated statistical analyses. This was done by Birnbaum et al. [77] who derived a 25-gene classifier for overall survival in resectable pancreatic cancer.

### Selective reporting and risk of bias

Reporting bias is a problem known for many years. In the context of diagnostic and prognostic studies, Rifai et al. [78] clearly stated that there is time for action, and a brief overview is given in a box entitled “*Selective reporting*” in the E&E paper of REMARK [5]. Ioannidis raised awareness for possible drivers for the lack of reliability of published biomedical research and the large number of false-positive results [79], including small sample sizes, small effect sizes, selective reporting of statistically significant results, or exploratory and hypothesis-generating research. This is also noted by Andre et al. [80] who discuss publication bias and hidden multiple-hypothesis testing distorting the assessment of the true value of markers. Hidden multi-hypotheses testing arises when several markers are tested by different teams using the same samples. The more hypotheses (i.e., marker association with outcome) that are tested, the greater the risk of false-positive findings. They stress the importance of a comprehensive marker study registry. Yavchitz et al. [81] identified 39 types of spin, which they classify and rank according to the severity. It is also known that many studies are started and that researchers do not finalize the study because they lose interest due to unsatisfactory early results. Empirical evidence of a “loss of interest bias” is given in [82]. In a systematic review of prognostic factors in oncology journals with an impact factor above 7, overinterpretation and misreporting were assessed in high-impact journals [9]. The authors identified misleading reporting strategies that could influence how readers interpret study findings. Doussau et al. [83] compared protocols and publications for prognostic and predictive marker studies. Not surprisingly, they found that protocols are often not accessible or not used for these studies and publications were often explicitly discordant with protocols.

In the section above, we referred to the critical appraisal of COVID prediction models by Wynants et al. [72]. Statements and the related editorial refer to the first publication of this “living systematic review” which included 232 prediction models in the third update. The authors had used the CHARMS checklist and assessed the risk of bias using PROBAST (Prediction Model Risk of Bias Assessment Tool) [70, 84]. The latter is organized into 4 domains: participants, predictors, outcome, and analysis. These domains contain a total of 20 signaling questions to facilitate structured judgment of risk of bias, which is defined to occur when shortcomings in study design, conduct, or analysis lead to systematically distorted estimates of model predictive performance. Wynants et al. [72] found that *All models reported moderate to excellent predictive performance, but all were appraised to have high risk of bias owing to a combination of poor reporting and poor methodological conduct for participant selection, predictor description, and statistical methods used*. We agree that the risk of bias has to be assessed as “high” if a study is badly reported. More detailed reporting would allow to assess the quality of the analysis and some of the 232 prediction models may have received a more positive assessment by Wynants et al [72].

### Barriers to better reporting, steps in the right direction, and more action needed

Above, we discuss that problems from single studies transfer to related meta-analyses and give several examples illustrating that the prognostic value of many markers is still unclear after more than a decade after the first publications, followed by hundreds of publications from other groups. Obviously, as for areas like treatment comparisons and (unbiased) estimate of treatment effects, evidence synthesis is also needed in prognosis research [85]. Debray et al. [85] discuss a number of key barriers of quantitative synthesis of data from prognosis studies. This includes lack of high-quality meta-data due to poor reporting of study designs, lack of uniformity in statistical analysis across studies, lack of agreement on relevant statistical measures, and lack of meta-analytical guidance for the synthesis of prognosis study data and emphasize also that there is relatively little guidance on how to do the actual meta-analysis of results from prognosis studies. They describe statistical methods for the meta-analysis of aggregate data, individual participant data and a combination thereof. The ideal would be the availability of individual participant data from all relevant studies. Such analyses become more popular and a review identified already 48 individual participants’ data MAs of prognostic factor studies published until March 2009. However, it is obvious that such projects face numerous logistical and methodological obstacles, and their conduct and reporting can often be substantially improved [86]. We refer to [87, 88] for more recent examples but there are several barriers for individual participant data meta-analysis studies [85, 89], and they are still rare exceptions in prognosis research. Meta-analyses based on aggregate data are common but can they provide suitable assessments of the value of prognostic markers? Obviously, inadequate reporting of the original studies is an important reason that the answer is a clear “no.” A number of other critical issues are briefly discussed by Sauerbrei and Haeussler [54].

There are several important steps which help to improve prognosis research. Starting in 2004, Richard Riley, Doug Altman, and several colleagues initiated the Cochrane Prognosis Methods Group [90]. The group brought together researchers and clinicians with an interest in generating the best evidence to improve the pathways of prognostic research and facilitate evidence-based prognosis results to inform research, service development, policy, and more [91, 92]. In 2010, Riley, Hemingway, and Altman formed the PROGRESS (PROGnosis RESearch Strategy) partnership [93]. This group published several papers about prognosis research, with a paper giving recommendations for improving transparency in prognosis research as the most relevant for this discussion [94]. A related book was published [95], including a chapter on “Ten principles to strengthen prognosis research” [96], some of the principles refer to specific issues of analyses but more guidance for analysis is needed. Providing accessible and evidence-based guidance for key topics in the design and analysis of observational studies is the main objective of the STRengthening Analytical Thinking for Observational Studies (STRATOS) initiative [97]. The topic group “Initial data analysis” emphasizes the importance of providing more details about the steps on the data of a study between the end of the data collection and the start of those statistical analyses that address research questions. In a recent review, they showed that early steps of analyses are often not mentioned and they provide recommendations for improvement [98]. Already in the REMARK E&E paper [5], it was stressed that data manipulations and pre-modeling decisions could have a substantial impact on the results and should be reported. Despite its importance, reporting of initial data analysis steps is usually not done.

Recently, Dwivedi and Shukla [99] proposed the statistical analysis and methods in biomedical research (SAMBR) checklist, but it needs to be seen whether this proposal finds wider acceptance. Anyhow, more generally accepted guidance for the design and analysis of prognostic factor studies would certainly help to standardize analyses and the quality of reporting would improve [92]. Several other relevant steps have been proposed, but adherence is still bad. Registration of prognosis studies and publishing protocols to reduce selective reporting, improve transparency, and promote data sharing was often proposed during the last decade [80, 94, 100, 101] but is hardly followed. Sauerbrei et al. [17] proposed that journals require a REMARK checklist for the first submission of a new paper. Such a checklist would help reviewers and editors in the submission process and also readers when checking for specific issues in a paper. A checklist would help authors to realize which parts of the analysis are missing or may need extensions. We refer to Tomar et al. [102] for a nice example but altogether this easy task to improve prognosis research is hardly used.

Further issues are discussed in a paper about Doug Altman as the driving force of critical appraisal and improvements in the quality of methodological and medical research. Sauerbrei et al. [92] summarize Doug Altman’s message concerning (1) education for statistics in practice, (2) reporting of prognosis research, (3) structured reporting and study registration, and (4) standardization and guidance for analysis. Using COVID-19 research as an example, Van Calster et al. [103] provide reliable and accessible evidence that the scandal of poor medical research, as denounced by Altman in 1994 [104], persists today. In three tables, they summarize *(1) issues which lead to research waste, (2) practices which result in prioritizing publication appearance over quality, and (3) examples of initiatives to improve the methodology and reproducibility of research*.

## Conclusions

We consider inadequate reporting of single studies as one of the most important reasons that the clinical relevance of most markers is still unclear after years of research and dozens of publications. As it is clear from the examples of inadequately reported studies, there is an urgent need to improve the completeness and reporting quality of all parts of the analyses conducted.

We propose to summarize the key information from a prognostic factor study in a structured profile, ideally prospectively created and registered. Defining all details of the analysis part when designing a study would correspond to a detailed statistical analysis plan. Obviously, an SAP may have to be modified, for example, if important assumptions are violated. Any such changes should be described in the paper’s corresponding REMARK profile and readers would then see all analyses and would be able to distinguish between preplanned analyses, data-dependent modifications, and additional subgroup or sensitivity analyses, if performed. Such a severe improvement in the reporting of single studies will have an impact on related systematic reviews and meta-analyses and therefore on the quality of prognosis research. The concept of structured reporting can be easily transferred to many other types of studies to improve reporting and transparency of analyses in medical and methodological research.

## Supplementary Information

**Additional file 1.**

## Data Availability

The data (our REMARK profiles) are published in the appendix.

## Abbreviations

BCRT: *Breast Cancer Research and Treatment*
CHARMS: Checklist for critical Appraisal and data extraction for systematic Reviews of prediction Modelling Studies
CI: Confidence interval
CONSORT: Consolidated Standards of Reporting Trials
E&E: Explanation and elaboration
EJC: *European Journal of Cancer*
HR: Hazard ratio
IJC: *International Journal of Cancer*
JCO: *Journal of Clinical Oncology*
MA: Meta-analysis
PRISMA: Preferred Reporting Items for Systematic Reviews and Meta-Analyses
PROBAST: Prediction Model Risk of Bias Assessment Tool
PROGRESS: PROGnosis RESearch Strategy
PTC: Papillary thyroid cancer
REMARK: Reporting Recommendations for Tumor Marker Prognostic Studies
ROC: Receiver operating curve
SAMBR: Statistical Analysis and Methods in Biomedical Research
SAMPL: Statistical Analyses and Methods in the Published Literature
SAP: Statistical analysis plan
STRATOS: STRengthening Analytical Thinking for Observational Studies
TRIPOD: Transparent Reporting of a multivariable prediction models for Individual Prognosis Or Diagnosis
