# Supplementary material for: Structured reporting to improve transparency of analyses in prognostic marker studies
Source: BMC Med. 2022 May 12;20:184. doi: 10.1186/s12916-022-02304-5 (PMC9095054; doi:10.1186/s12916-022-02304-5)
Supplement: Supplementary file 1 — Additional file 1. [file 12916_2022_2304_MOESM1_ESM.pdf]

Supplementary to

Structured reporting to improve transparency of analyses in prognostic marker studies

Willi Sauerbrei[1]*, Tim Haeussler[1], James Balmford[1]^ and Marianne Huebner[2]

[1] Institute for Medical Biometry and Statistics, Faculty of Medicine and Medical Center – University of Freiburg, Freiburg, Germany
[2] Department of Statistics and Probability, Michigan State University, East Lansing, MI, USA

^James Balmford is deceased

*Correspondence: wfs@imbi.uni-freiburg.de

Hayashi et al (2015) Prognostic factors of HER2-positive breast cancer patients who develop brain metastasis: a multicenter retrospective analysis. *Breast Cancer Res. Treat.* **149**: 277–284, doi:10.1007/s10549-014-3237-7

A. Patients, treatment, and variables

Patients: HER2+ breast cancer with brain metastasis, 04/2001-12/2012, data from 24 of 34 institutions at Japan Clinical Oncology Group, Breast Cancer Study Group	
**1466**	Patients assessed (brain metastasis as first recurrence or developed brain metastases during systemic treatment, HER-2 positive)
**1034**	Patients excluded (see ref. 5 – data collected for 1466 patients initially)
**432**	Patients included for analysis
Follow-up: Median follow-up 50.6 months	
Marker:	M1=Estrogen receptor (ER) status
Outcomes (Events):	OS (unknown number of events), BMFS (all 432, no results presented here, but refers to a previous paper by the authors)
Variables:	v1=symptoms of brain metastases, v2=number of brain metastases (≤3/>3), v3=histological grade (G1/G2-3)
Treatment/variables:	Time dependent treatments, for details see second paragraph of 'clinical characteristics' and Tab.2, t1=trastuzumab before development of brain metastases, t2=trastuzumab after development of brain metastases, t3=lapatinib after development of brain metastases

Abbreviations: OS=overall survival, time from diagnosis of brain metastasis to death, censored at last follow-up
BMFS=brain metastasis-free survival, time from breast cancer diagnosis to brain metastasis diagnosis or last follow-up

B. Statistical analysis of survival outcomes

Aim	n	Outcome (Events)	Variables considered	Results/ remarks
A1: Effect of treatment	432	OS (?)	t2, t3	Kaplan-Meier estimate, HR, CI, p-value, Fig. 1, Tab. 2
A2: Combined effect of treatment	432	OS (?)	t2, t3	Kaplan-Meier estimate, HR, CI, p-value, Fig. 2
A3: Univariate	Varies due to missing	OS (?)	M1, v1-v3, t1-t3	HR, CI, p-value, Tab. 2
A4: Multivariable	Unknown due to missing	OS (?)	v2, t2, t3[1]	HR, CI, p-value, Text and Tab. 2
Statistical software packages used:		SPSS v.21		

[1] Unclear whether A4 included only these 3 variables or all variables listed at A3.

Huzell L et al (2015) History of oral contraceptive use in breast cancer patients: impact on prognosis and endocrine treatment response. *Breast Cancer Res. Treat.* **149**: 505–515, doi:10.1007/s10549-014-3252-8

A. Patients, treatment, and variables

Patients: Diagnosis of primary breast cancer 2002-2011 at Skåne University Hospital Lund, Sweden	
**1045**	Patients assessed
**51**	Patients excluded (treatment prior to surgery)
**994**	Patients included for descriptive analysis
**46**	Patients additionally excluded (in situ carcinoma: 38; metastatic spread within 3 months: 8)
**948**	Patients included for predictive analysis of risk of an early breast cancer (BC) event
Treatment and follow-up: Standard care. Follow-up up to 9 years (until December 2012); median 3.03 years (IQR 1.93-5.23)	
Markers:	Oral contraceptive (OC) use:<br>M1 = Ever OC use (yes/no)<br>M2 = Use before age 20 (yes/no)<br>M3 = OC use before first child (yes/no)<br>M4 = OC start 1974 or later (proxy for dose) (yes/no)<br>M5 = Duration of OC use (continuous)
Outcomes (events):	Breast cancer events (BCE) (100): distant metastasis (DM) (65),
Variables:	v1 = age, v1_c50 = age ≥50 (proxy for menopause), v2 = tumor size[1], v3 = grade[2], v4 = nodal involvement, v5 = hormone receptor status, v6 = BMI[3], v7 = endocrine treatment
Missing data:	See Tables 1 and 2

Abbreviations: BMI=body mass index, Breast cancer event=local or regional recurrence, distant metastasis or contralateral breast cancer

[1] Invasive tumor size ≥21 or muscle or skin involvement (yes/no) in multivariable model
[2] Grade I-II vs grade III in multivariable model
[3] BMI ≥25 kg/m^2 (yes/no) in multivariable model

B. Statistical analysis of survival outcomes

Aim	n	Events	Outcome	Variables considered	Results/ remarks
IDA1: Data screening and definitions of categories	994	NA		M1, M2, M3	Stat. methods. Definition of markers (categorization of OC use)
IDA2: descriptive	994	NA	OC use categories (M1,M2,M3)	v1-6, plus 12 descriptive-only variables	Table 1 (patient characteristics), Table 2 (tumor characteristics)
A1: Multivariable	948	100	BCE	M1, M2, M3, v1-v6	Reported only in text (no data provided), p.508 first column
A2: Univariate/Multivariable subgroup v1_c50 ≥ 50	760	70	BCE	M1, M2, M3, v1-v6	For M2: Fig 2a, Tab 3. Kaplan-Meier, Log-rank and HR. M1 and M3 non-significant and reported only in text, p.508 first column
A3: Univariate/Multivariable subgroup v1_c50 < 50	188	30	BCE	M1, M2, M3, v1-v6	For M2: Fig 2b, Tab 3. Kaplan-Meier, Log-rank and HR. M1 and M3 non-significant and reported only in text, p.508 first column
A4: Multivariable subgroup v1_c50 ≥ 50	?	?	DM	M2; v1-v6	Reported in text, no data provided: p.508 second column
A5: Multivariable subgroup v1_c50 < 50	?	?	DM	M2; v1-v6	Log-rank and HR in text, p.508 second column
A6: Multivariable	948	100	BCE	M4; v1-v6	HR in text, p.508 second column
A7: Multivariable subgroup v1_c50 ≥ 50	760	70	BCE	M4, M5; v1-v6	HR in text, p.508 second column
A8: Multivariable subgroup v1_c50 < 50	188	30	BCE	M4, M5; v1-v6	HR in text, p.508 second column
A9: Multivariable subgroup v7 (TAM treatment), v1_c50 (age ≥ 50), v5 (ER+)	372	29	BCE	M1; v1-v7	Fig 3a. Kaplan-Meier, Log-rank and HR - adjusted for tumor and patient characteristics, and aromatase inhibitor (AI) treatment
A10: Multivariable subgroup v7 (AI treatment), v1_c50 (age ≥ 50), v5 (ER+)	277	26	BCE	M1; v1-v7	Fig 3b. Kaplan-Meier, Log-rank and HR - adjusted for tumor and patient characteristics, and tamoxifen (TAM) treatment
Statistical software packages used:			SPSS v.19		

Jerzak KJ et al (2015) Thyroid hormone receptor α in breast cancer: prognostic and therapeutic implications. *Breast Cancer Res. Treat.* **149**: 293–301, doi:10.1007/s10549-014-3235-9

A. Patients, treatment, and variables

Patients: Primary invasive breast cancer (≥ 1cm in diameter) in 2007, tissue samples from the pathology archive, Hamilton Health Sciences, Canada	
**?**	Patients assessed
**?**	Patients excluded (no adequate tissue was available; treated with neoadjuvant therapy; multifocal tumors; medical history not available; known BRCA mutation)
**129**	Patients included for analysis[1]
Treatment and follow-up: Mastectomy or segmental breast resection; minimum follow-up = 5 years	
Markers:	M1 = THRα1 (continuous/binary), M2= THRα2 (continuous/binary)
Outcomes (events):	OS (22), RFS (36)
Further variables:	v1 = Age (at diagnosis), v2 = tumor size, v3 = nodal stage, v4 = T stage, v5 = stage, v6 = grade, v7 = ER status, v8 = PR status, v9 = HER2 status, v10 = lymphovascular invasion, v11 = mitotic count, v12 = hypothyroid, v13 = chemotherapy, v14 = hormone therapy
Missing data:	See Table 1

B. Statistical analysis of survival outcomes

Aim	n	Events	Outcome	Variables considered	Results/ remarks
IDA: Univariate Determine cutpoints M1 and M2	129	22	OS	M1, M2	Stat methods, p.297 results. Action: no optimal cutpoint identified. Allred score ≥6 used instead.
A1: Univariate	Varies, 129 for M1/M2	36/22	RFS, OS	M1-2, v1-14	Tab 3 (RFS) and Tab 4 (OS), Results. M1 and M2 were analysed continuous, dichotomized, or as a ratio.[2]
A2: Univariate	129	22	OS	M2 (bin)	Results. K-M plot, Fig 5a
A3: Multivariable forward selection (FS)	128	36	RFS	M1, M2, v1, v2, v4, v6-v12	Tab 3. Units and/or transformations shown in table. Not reported in text.

[1] The paper states that the study sample was n=130, however data on the markers M1 and M2 were only available for 129. See Table 1.
[2] Multivariate HR for M2 (cont.) from A3 was mistakenly reported in text.

A4: Multivariable FS	113	?	RFS	M1,M2, v1, v2-v4, v6-v12	Tab 3. Units and/or transformations shown in table. Not reported in text.
A5: Multivariable FS	129	22	OS	M1,M2, v1, v2, v4, v6-v12	Tab 4. Units and/or transformations shown in table. Not reported in text.
A6: Multivariable FS	110	?	OS	M1,M2, v1, v2-v4, v6-v12	Tab 4. Units and/or transformations shown in table. Not reported in text
A7: Multivariable	129	36/22	RFS, OS	M2, v7, others?	HR, CI, p-value p.299. Only reported in text
A8: Univariate M1, M2 dichotomized combinations	129	22	OS	M1,M2	Fig 5b
Statistical software packages used:			No information given		

Abbreviations: NA=not applicable, NR=not reported, OS=overall survival, RFS=recurrence-free survival

Billingsley et al (2015) Polymerase ε (POLE) mutations in endometrial cancer: clinical outcomes and implications for Lynch syndrome testing. *Cancer* **121**: 386–394, doi:10.1002/cncr.29046

A. Patients, treatment, and variables

Patients: Matched normal and cancer tissue samples from endometrial cancer patients prospectively collected at time of hysterectomy surgery at Washington University (St Louis, MO)	
**544**	Patient tissue samples collected
**9**	Patients excluded due to unsuccessful molecular analysis of tissues
**535**	Patients included for analysis
Treatment and follow-up: Hysterectomy; references to several earlier publications; unknown years of surgery; median follow-up 68.4(somatic mutation)/71(wild type) months	
Markers:	M1=Polymerase ε (POLE) mutational status (somatic mutation vs wild type POLE)
Outcomes (Events):	PFS (unknown number of events) OS (unknown number of events)
Further variables:	v1=age (<60/≥60), v2=stage, v3=grade, v4=LVSI, v5=depth of invasion, v6=adjuvant therapy, v7=BMI, v8=race
Missing data:	Ranged from 0-55 cases for the above variables; complete data only for v1 (Footnote table 2)

Abbreviations: PFS=progression free survival, OS=overall survival, LVSI=lymphovascular space involvement, BMI=body mass index

B. Statistical analysis of survival outcomes

Aim	n	Outcome (Events)	Variables considered	Results/ remarks
IDA: Comparison of features between POLE mutations and wild type	30 (mutation)/ 505 (wild type)	-	M1,v1-v8	Distribution of v1-v8, p value of associations M1 with v1-v8, Tab 2
A1: Univariate	Varies due to missing data	PFS (?), OS (?)	M1, v1-v7	HR, p-value in Tab 3, Kaplan-Meier for M1 in Fig 2. OS<PFS. Probably an error in the caption.
A2: Multivariable (incl. v from A1 with p<0.1)	Unknown due to missing data	PFS (?)	v1-v6	HR, p-value, CI, Tab. 4. M1 not included. In univariate analysis p>0.10
A3: Multivariable (incl. v from A1 with p<0.1)	Unknown due to missing data	OS (?)	M1, v1-v7	HR, p-value, CI, Tab. 4
A4: Multivariable stepwise (incl. v from A1 with p<0.1)	Unknown due to missing data	PFS (?)	v2,v3,v4	HR, p-value, CI, Supporting Tab. 2
A5: Multivariable stepwise (incl. v from A1 with p<0.1)	Unknown due to missing data	PFS (?), OS (?)	v1,v2,v3,v4	HR, p-value, CI, Supporting Tab. 2
Statistical software packages used:	SAS 9.2; STATA SE 10			

Huang et al (2015) Prognostic value of pretreatment circulating neutrophils, monocytes, and lymphocytes in oropharyngeal cancer stratified by human papillomavirus status. *Cancer* **121**: 545–555, doi:10.1002/cncr.29100

A. Patients, treatment, and variables

Patients: Oropharyngeal cancer patients with known HPV status 2000-2010; University of Toronto, Canada	
**1108**	Patients assessed (oropharyngeal cancer, treated with primary chemoradiotherapy)
**406**	Patients excluded (298 HPV unknown; 108 pretreatment complete blood counts unavailable)
**702**	Patients included for analysis (510 HPV+, 192 HPV-)
Treatment and follow-up: Primary radiotherapy/chemoradiotherapy; median follow-up 5.1 years for HPV+ and 4.1 years for HPV-	
Markers:	M1=neutrophil count (CNC), M2=lymphocyte count (CLC), M3=monocyte count (CMC)
Outcomes (Events):	RFS (analyzed as competing risks, 114 HPV+ and 77 HPV-), OS (136 HPV+ and 121 HPV-)
Further variables:	v1=age (cont), v2=smoking pack years (>10, ≤10), v3=T classification, v4=N classification, v5=treatment (RT vs RT/CRT), v6=sex, v7=subsite,v8=smoking status

Abbreviations: RFS=recurrence free survival, OS=overall survival, HPV=human papilloma virus, OPC= oropharyngeal cancer, RT=radiotherapy, CRT=chemoradiotherapy

B. Statistical analysis of survival outcomes

Aim	n	Outcome (Events)	Variables considered	Results/ remarks
IDA: Associations of variables with markers separately by HPV status	HPV+ : 510 HPV- : 192	-	M1, M2, M3, v2	Results, Fig. 1
A1: Cutpoints of markers determined by median; associations with variables	510/192		M1, M2, M3, v1-v8	Results, Tab. 1 and 2
A2: Univariate, M1-3 (bin)	510 HPV+	RFS (114), OS (136)	M1-3	HR, CI, p-value, Results, Fig. 2
A3: Univariate, M1-3 (cont)	510 HPV+	RFS (114), OS (136)	M1-3, v1-v5	HR, CI, p-value, Tab. 3
A4: Multivariable	510 HPV+	RFS (114), OS	M1-3, v1-	HR, CI, p-value,

M1-3 (cont)		(136)	v5	Results, Tab. 3, C index
C1: Check for proportional hazards[1]	510 HPV+	RFS (114), OS (136)	M1-3, v1-v5	“No violation identified” (text p.548)
A5: Univariate, M1-3 (bin)	192 HPV-	RFS (77), OS (121)	M1-3	HR, CI, p-value, Results, Fig. 3
A6: Univariate, M1-3 (cont)	192 HPV-	RFS (77), OS (121)	M1-3, v1-v5	HR, CI, p-value, Tab. 3
A7: Multivariable M1-3 (cont)	192 HPV-	RFS (77), OS (121)	M1-3, v1-v5	HR, CI, p-value, Results, Tab. 3
Statistical software packages used:	No information given			

[1] Not stated whether this was also carried out in the HPV- group

Price et al (2015) Does the primary site of colorectal cancer impact outcomes for patients with metastatic disease? *Cancer* **121**: 830–835, doi:10.1002/cncr.29129

A. Patients, treatment, and variables

Patients: Metastatic colorectal cancer (mCRC) data from South Australian mCRC registry, Feb. 2006-Feb. 2013	
**?**	Patients assessed
**?**	Patients excluded
**2972**	Patients in the registry and included for analysis. Unclear whether others were assessed and not included in the registry
**Subgroups**	a=all patients, b=liver resection without chemotherapy (n=57), c=chemotherapy/systemic therapy ±metastasis resection (n=1738), d=liver resection ±chemotherapy (n=123[1]), e=chemotherapy/systemic therapy only (n=?)
Treatment and follow-up: Liver resection and/or chemotherapy, or no active treatment. Follow-up (range or median) not reported; some information provided in the Kaplan-Meier plots	
Markers:	M=primary colorectal site (binary: left sided including rectum/right sided), M(3)=primary colorectal site (3 groups: left/right/rectum), M(10)=exact anatomical primary site (10 groups)
Outcomes (events):	OS[2] (unknown)
Further variables:	v1=sex, v2=age (cont.), v3=stage at diagnosis (binary), v4=grade (4 cat.), v5_1[3]=metastatic site - liver only, v5_2=lung only, v5_3=liver and lung, v5_4=liver and other (not lung), v5_5=lung and other (not liver), v5_6=other, v6=chemotherapy (y/n), v7=liver surgery (y/n), v8=clinical trials, v9=KRAS exon 2 mutation (y/n)

Abbreviations: OS=overall survival, mCRC= metastatic colorectal cancer

B. Statistical analysis of survival outcomes

Aim	n	Outcome (Events)	Variables considered	Results/ remarks
A0: Patient characteristics by M, M(3) and M(10)	Varies due to missing values	-	M, v1-v8	p-values, Tab. 1 for M: v9 not included in Table 1 but discussed in results
A1: Effect of M in all patients, univariate	2972	OS (?)	M	HR (+CI), p-value, Tab. 2
A2: Effect of M in all patients, multivariate	? missing ness	OS (?)	M, v1-v7	HR (+CI), p-value, Tab. 2; Kaplan-Meier estimate, Numbers at risk, Fig. 1a
A3: Survival in subgroup b	57	OS (?)	M	5-year median OS, p-value

[1] Figure 2 however suggests n=414.
[2] OS is undefined, and no information about censoring is provided, other than to say that the final censor date was Feb 12, 2013.
[3] All three of liver, lung and other was probably the referent.

A4: Survival in subgroup c	1738	OS (?)	M	Kaplan-Meier estimate, HR (+CI), p-value, Numbers at risk, Fig. 1b
A5: Survival in subgroup d	123	OS (?)	M	Kaplan-Meier estimate, HR (+CI), p-value, Numbers at risk, Fig. 2
A6: Survival in subgroup e	?	OS (?)	M	Median OS, p-value
A7: Effect of M(3) in subgroup c[4]	1738	OS (?)	M(3)	'Kaplan-Meier estimate, HR (+CI), p-values, Numbers at risk, Fig. 3
A8: Univariate by M(10)	2972	OS	M(10)	Rate of median OS by M(10)
A9: Univariate by M(10) subgroup c[5]	1738	OS	M(10)	Rate of median OS by M(10)
Statistical software packages used:			Stata 13.0	

[4] Results are not shown or discussed for subgroups b, d and e

[5] Results are not shown or discussed for subgroups b, d and e

Gonzalez-Vallinas et al (2015) Clinical relevance of the differential expression of the glycosyltransferase gene GCNT3 in colon cancer. *Eur J Cancer* **51**: 1–8, doi:10.1016/j.ejca.2014.10.021

A. Patients, treatment, and variables

Patients: FFPE[1] samples from curative resection of colon cancer patients at La Paz University Hospital, Madrid		
**?**	Patients assessed (age ≥18, stage II primary colon cancer, follow-up ≥ 36 months, high quality RNA sample - 3/1 excluded in training/validation series)	
**?**	Patients excluded (incomplete excision, death within 30 days of surgery, mixed histological features, other cancers in prev. 5 years, inflammatory bowel disease, hereditary tumours)	
**77/119**	Patients included for analysis (training series/validation series)	
**18**	Healthy tissue samples	
Treatment and follow-up: FFPE samples from curative resection from 2000-04 (training series, median follow-up 72 months) and 2005-08 (validation series, median follow-up 42 months)		
Markers:	M1=GCNT3 expression (dichotomized)	
Outcomes (Events):	Disease-free survival (DFS) (22/18)[2]	
Further variables:	v1=age (≤70/>70), v2=localisation, v3=grade, v4=sex, v5=no. of lymph nodes, v6=T stage, v7=chemotherapy, v8=vascular invasion, v9=perineural invasion	

DFS: recurrence as only event mentioned

B. Statistical analysis of survival outcomes

Aim	n (training/ validation series)	Outcome (Events)	Variables considered	Results/ remarks
A1: Comparison to healthy tissue	77/119 18 healthy	-	M1(continuous)	p-value, t-test, Fig. 1
A2: Determine 'optimal' cutpoint in training series	77	DFS (22)	M1 (continuous)	c-index using 100 times 5-fold cross-validation, cutpoint set to 2.75 Q[3]
A3: Univariate, training series	77	DFS (22)	M1	Kaplan-Meier-estimate, p-value, Fig. 2a; HR, CI, Tab. 2
A4: Univariate, validation series	119	DFS (18)	M1	Kaplan-Meier-estimate, p-value, Fig. 2b; HR, CI, Tab. 2
IDA: Correlation to M1, univariate, validation series	Varies due to missing data		M1, v1-v9 (probably)	v6, v8, v9 significant ($p<0.05$), p.4 second column
A5: Multivariable, training series	?	DFS (?)	M1, v1, v6, v8, v9	HR, p-value, CI, Tab. 2. Age + sign. variables in IDA

[1] FFPE = Formalin-fixed, paraffin-embedded.
[2] Colon cancer-specific death also quantified, however not examined in any analyses.
[3] Q = Quantification of GCNT3 expression, calculated with the $2^{-\Delta Ct}$ method.

A6: Multivariable, validation series	119	DFS (18)	M1, v1, v6, v8, v9	HR, p-value, CI, Tab. 2
Statistical software packages used:			R v. 2.15 (survival)<br>SPSS v.20 (t-test)	

Hokuto D et al (2015) Clinical impact of herpesvirus entry mediator expression in human hepatocellular carcinoma. *Eur J Cancer* **51**: 157–165, doi:10.1016/j.ejca.2014.11.004

A. Patients, treatment, and variables

Patients: Hepatocellular carcinoma surgery, 2000-2012 at Nara Medical University, Japan	
**?**	Patients assessed (surgery for HCC between 2000 and 2012)
**?**	Patients excluded
**150**	Patients included for analysis
Treatment and follow-up: Surgical resection. Follow-up until Sep. 2013 (median 51.8 months, range 6.7 to 151.7)	
Markers:	M = HVEM expression (cont. variable, dichotomized by cutoff at 50% stained)
Outcomes (Events):	RFS (86), OS (?), IHR (78), EHR (36), Early recurrence (within 2 years after surgery, 51)
Further variables:[1]	v1 = age*, v2 = gender, v3 = cirrhosis, v4 = viral status, v5 = AFP level*, v6 = PIVKA-II*, v7 = tumour size (<5cm/≥5cm), v8 = tumour number (single/multiple), v9 = histol. differentiation (well/moderate or poor), v10 = vascular invasion, v11 = capsule invasion, v12 = TNM stage (I/II-IV), v13=alcohol consumption

*dichotomized at the median
Abbreviations: IHR/EHR = intra-/extra-hepatic recurrence

B. Statistical analysis of survival outcomes

Aim	n	Outcome (Events)	Variables considered	Results/ remarks
IDA1: Correlation	150	-	M, v1-v13	p-values for M (binary) with Vx; χ^2 or t-test, Tab. 1
IDA2: Cutpoints at median			v1,v5,v6	Stat methods
A1: Univariate	150	RFS (86), OS (?)	M	Kaplan-Meier estimates, p-values, Fig. 2
A2: Univariate	150	RFS (86), OS (?)	M, v1-v12	HR, CI, p-values, Tab. 2
A3: Multivariate	150	RFS (86), OS (?)	M, v6, v7, v10	HR, CI, p-values, Tab. 3, variable selection unclear
A4: Univariate	150	IHR (78), EHR (36), early recurrence (51)	M	Chi-square p-values, Tab. 4
Statistical software packages used:		No information given		

[1] All variables binary.

Thurner et al (2015) The elevated C-reactive protein level is associated with poor prognosis in prostate cancer patients treated with radiotherapy. *Eur J Cancer* **51**: 610–619, doi:10.1016/j.ejca.2015.01.002

A. Patients, treatment, and variables

Patients: Treated for primary prostate cancer at the Department of Therapeutic Radiology and Oncology, Medical University of Graz, Austria, 2003-2007	
**>700**	Patients assessed
**>439**	Patients excluded (Did not meet below criteria, as well as those with a follow-up of < 4 months)[1]
**261**	Patients included for analysis (histologically confirmed primary prostate cancer + pre-treatment CRP levels taken)
Treatment and follow-up: 3D radiation therapy in curative intent; median follow-up 80 months	
Markers:	M = pre-treatment CRP (continuous variable; analyses for dichotomized or categorical data, based on optimal cutpoints)
Outcomes (events):	CSS – primary outcome (24), OS (59), DFS (56)
Further variables:	v1 = age at diagnosis, v2 = PSA at diagnosis, v3 = tumor stage, v4 = Gleason score, v5 = risk group[2], v6 = total duration of ADT

Abbreviations: CRP=C-reactive protein, CSS=Cancer-specific survival: time from diagnosis to date of prostate cancer related death, OS=Overall survival, DFS=Clinical disease-free survival, ADT=androgen deprivation therapy

B. Statistical analysis of survival outcomes

Aim	n	Outcome (Events)	Variables considered	Results/ remarks
IDA1: Correlations	Varies[3]		M, v1-v5	Results p.613 first column
IDA2: Determination of optimal cutpoint for M	261	CSS (24)	M	CRP dichotomised into high (≥ 8.6 mgl^{-1}) and low (< 8.6 mgl^{-1})
A1: Univariate survival analysis	261	A1.1 CSS (24)<br>A1.2 OS (59)<br>A1.3 DFS (56)	M	Kaplan-Meier-estimates, figures 1-3
A2: Univariate associations	Varies	A2.1 CSS (24)<br>A2.2 OS (59)<br>A2.3 DFS (56)	M, v1, v2, v3, v4, v6	HR, CI, p-values, Tab. 2-4
A3: Multivariate (incl. v. from A2.1, A2.2 and A2.3 with p<.05)	?	CSS (?)<br>OS (?)<br>DFS (?)	M, v2, v3, v4, v6	HR, CI, p-values, Tab. 2 (CSS), Tab. 3 (OS), Tab. 4 (DFS)
A4: Univariate, high risk (v5)	144	A4.1 CSS (?)<br>A4.2 OS (?)<br>A4.3 DFS (?)	M	HR, CI, p-value, Tab. 5

[1] It is not stated how many patients had a follow-up of < 4 months, nor whether these were excluded prior to the final 261 or were excluded from the 261 in subsequent analyses. We will assume the former.
[2] Three categories; see ref. 31.
[3] Due to missing data. Numbers are available in Table 1.

A5: Multivariate, high risk (v5)	144	A5.1 CSS (?)<br>A5.2 DFS (?)	M, v6	HR, CI, p-value, Tab. 5[4]
A6: Univariate, intermediate risk (v5)	66	A6.1 CSS (?)<br>A6.2 OS (?)<br>A6.3 DFS (?)	M	p values in text, p.615 first column (all n.s.)
A7: Univariate, low risk (v5)	51	A7.1 CSS (?)<br>A7.2 OS (?)<br>A7.3 DFS (?)	M	p values in text, p.615 first column (all n.s.)
IDA3: Cutpoint determination for M in subgroups of v5	261	CSS (24)	M	CRP categorised with cut-off values of 8.9, 8.4 and 13.4 for the v5 risk groups
A8: Univariate by v5 subgroups	144/66/51	A8.1 High-risk, CSS (?)<br>A8.2 Intermediate-risk, CSS (?)<br>A8.3 Low-risk, CSS (?)	M	No data shown, p.615 first column (findings same as A4.1, A6.1 and A7.1)
IDA4: Cutpoint determination for M in subgroups of v6[5]	261	CSS (24)	M	CRP dichotomised with cut-off values of 6.7 and 8.9 for patients with and without ADT
A9: Univariate by v6 subgroups	?/?	A9.1 CSS (?)<br>A9.2 OS (?)<br>A9.3 DFS (?)	M	HR, CI, p-value, p.615 first and second columns
Statistical software packages used:			SPSS v.20	

[4] No multivariate analysis was carried out for OS because v6 was not significant at A4.

[5] Here defined as with/without ADT

Keck et al (2015) Neuropilin-2 and its ligand VEGF-C predict treatment response after transurethral resection and radiochemotherapy in bladder cancer patients. *International Journal of Cancer* **136**: 443–451, doi:10.1002/ijc.28987

A. Patients, treatment, and variables

Patients: Bladder cancer patients (cN0M0 tumors) at Universitätsklinikum Erlangen between 1982-2007	
**473**	Patients assessed
**226**	Patients possibly excluded (see ref. 4 "same cohort" - 473 patients) due to missing data?
**247**	Probably patients with complete data (M1, M2, v1-v10) included for analysis
Treatment and follow-up: Bladder-sparing therapy (TURBT); follow-up time up to 15 years	
Markers:	M1 = NRP2 expression, M2 = VEGF-C expression, M3 = M1 and M2 combined
Outcomes (events):	OS(number of events unknown), CSS(number of events unknown)
Further variables:	v1=age, v2=sex, v3=tumor stage, v4=tumor grade, v5=resection status, v6=lymphatic vessel invasion, v7=blood vessel invasion, v8=carcinoma in situ, v9=multifocal tumor occurrence, v10=histologic subtype
Treatment in addition to TURBT:	T = RCT (subgroup t1, n=198) or RT only (subgroup t2, n=49)

Abbreviations: CSS=Cancer specific survival, TURBT=Transurethral resection of bladder tumor, RCT=Radiochemotherapy, RT=Radiotherapy only

B. Statistical analysis of survival outcomes

Aim	n	Outcome (no. of events never known!)	Variables considered	Results/ remarks
A1: Univariate, in 7 subgroups defined by combinations of v3 and T	varies by subgroup	OS, CSS	M1-M3	p-values, Tab. 2
A2: All patients	247	OS, CSS	M1-M3	Kaplan-Meier estimates, p-values (log-rank test), Fig. 2
A3: Subgroup t1	198	OS, CSS	M1-M3	Kaplan-Meier estimates, p-values (log-rank test), Fig. 3
A4: Subgroup t2	49	OS, CSS	M1-M3	Kaplan-Meier estimates, p-values (log-rank test), Supporting Inf. Fig. S1
A5: Univariate all patients	247	OS, CSS	M1-M3	RR[1], CI, p-values, Tab. 3
A6: Univariate subgroup t1	198	OS, CSS	M1-M3	RR, CI, p-values, Tab. 3

[1] A5-A8: Labelled as relative risk in Table 3, but should be a hazard ratio

A7: Multivariate all patients	247	OS, CSS	M1-M3, adjusted for v1-v10, T	RR, CI, p-values, Tab. 3, Text
A8: Multivariate subgroup t1	198	OS, CSS	M1-M3, adjusted for v1-v10	RR, CI, p-values, Tab. 3, Text
Statistical software packages used:	SPSS v.21			

Rödel et al (2015) Human papillomavirus DNA load and p16INK4a expression predict for local control in patients with anal squamous cell carcinoma treated with chemoradiotherapy. *International Journal of Cancer* **136**: 278–288, doi:10.1002/ijc.28979

A. Patients, treatment, and variables

Patients: Anal squamous cell carcinoma (SCC) treated at University Hospitals Frankfurt and Göttingen (time period?)	
**?**	Patients assessed (histological proof, curative intent of 5-FU-based chemoradiotherapy (CRT))
**?**	Patients excluded
**95**	Patients included for analysis, patients with data for HPV16 DNA load (n=80)
Treatment and follow-up: Homogeneously with standard CRT; median follow-up 40 months (range 1-264 months)	
Markers:	M1 = HPV16 DNA load*, M2 = $p16^{INK4a}$ expression**, M3 = combining dichotomized versions of M1 and M2***
Outcomes (events):	incidence of local failure (IL, n=21), incidence of distant metastases (ID, n=14), OS (n=27), CSS (n=16)
Further variables:	v1=age, v2=gender, v3=HIV status, v4=T-stage, v5=N-stage, v6=grading, v7=HPV genotype

*dichotomized at the median
**score system (see text paragraph "Immunohistochemical…" p. 280), dichotomized
***partly 2 categories (see Supp. tab. 1) and partly 3 categories (see Fig. 4)
Abbreviations: OS=time from start of CRT to death from all causes, CSS = time from start of CRT to cancer related death

B. Statistical analysis of survival outcomes

Aim	n	Outcome (Events)	Variables considered	Results/ remarks
IDA1: Distribution of v7, dependencies in 2x2 tables	95 (M2) 80 (M1)		M1, M2, v1-v7	p-values, Tab. 1, Tab. 2
IDA2: Define categories for M1	95		M1	Results. Use median. Low/high
IDA3: Define categories for M1 and M2	95(M2), 80 (M1)		M1, M2	Methods: Low/intermediate/high (see last paragraph p.280)
A1: Univariate[1]	95 (M2) 80 (M1)	IL (21), ID (14), CSS (27), OS (16)[2]	M1, M2, v1-v6	p-values, Kaplan-Meier estimates, 5y and 10y cumulative incidence of IL and ID in text, Tab. 3, Figs 2 and 3

[1] One missing value for v4 and six for v5; exact number of events unknown for analyses involving these variables.
[2] Events unknown for M1 however due to the exclusion of 15 cases

A2: Multivariable[3]	80[4]	IL (?)	M1, M2, v2, v4, v5	p-values, HR, CI, Tab. 3
A3: Multivariable	89	ID (?)	v2, v4, v5	p-values, HR, CI, Tab. 3
A4: Multivariable	89	CSS (?)	v2, v4, v5, v6	p-values, HR, CI, Tab. 3
A5: Multivariable	80	OS (?)	M1, v2, v4, v5	p-values, HR, CI, Tab. 3
A6: Combined marker M3, univariate	80	IL (?), ID (?), CSS (?), OS (?)	M3, v1-v6	p-values, Kaplan-Meier-estimates, Fig. 4, Supp. tab. 1
A7: Combined marker M3, multivariable	80	IL (?), CSS (?), OS (?)	M3, v1-v6	p-values, HR, CI, text, Supp. tab. 1
A8: Alternative cutpoints for M1 and M2	95 (M2) 80 (M1)	IL (?), ID (?), CSS (?), OS (?)[5]	M1, M2	Kaplan-Meier estimates, p-values, text (end of "Results"), Supp. tab. 2, Supp. fig
Statistical software packages used:		SPSS v.19		

[3] Analyses A2-A5 only include significant variables from A1.
[4] Minus an unknown number of missing cases; same for A5, A6 and A7.
[5] 5-year survival rates for each outcome.

Schirripa M et al (2015) Role of NRAS mutations as prognostic and predictive markers in metastatic colorectal cancer. *Int. J. Cancer* **136**: 83–90, doi:10.1002/ijc.28955

A. Patients, treatment, and variables

Patients: Tissue samples from patients with metastatic colorectal cancer (mCRC) from 2009-2012 were analysed at the pathology department of the University Hospital of Pisa	
**?**	Patients with available KRAS, BRAF and NRAS mutational status included
**?**	Patients excluded
**786**	Patients included for analysis
Treatment and follow-up: Follow-up not mentioned	
Markers:	M1=NRAS mutation (y/n), M2=KRAS mutation (y/n), M3=BRAF mutation (y/n), M4=all wt (no NRAS, KRAS or BRAF mutation)(y/n)[1]
Outcomes (events):	OS (?), PFS (?)
Further variables:	v1=sex, v2=age at diagnosis, v3=ECOG PS (0/1-2), v4=primary tumor site (nominal), v5=mucinous histology (y/n), v6=tumoral penetration (pT)(1-2/3-4), v7=nodal involvement (pN)(0/1-2), v8=time to metastasis (mts)(binary), v9=number of mts (1/>1), v10=resected primary (y/n), v11=liver only mts (y/n), v12=liver mts (y/n), v13=lung mts (y/n), v14=nodes mts (y/n), v15=peritoneal mts (y/n), v16=bone mts (y/n), v17=metastasis site (v11-v16) classified into 6 categories; see Table 2

Abbreviations: OS=overall survival (time from diagnosis of metastatic disease to death of al causes), PFS=progression free survival (time from beginning of treatment to disease progression or death of any cause)

B. Statistical analysis of survival outcomes

Aim	n	Outcome (Events)	Variables considered	Results/ remarks
IDA: Homogeneity	(786) various n due to missing	-	M1-M4, v1-v9, v11-v17	p-values, Tab. 1- 2, and various subgroup analyses reported in text only (p. 85)
A1: Univariate	786	OS (?)	M1- M4	Kaplan-Meier-estimate, Log-rank-test (p-value) Fig. 1
A2: Univariate	321 (47 (M1) + 274 (M4) see Tab.1)	OS (?)	M1, M4	Kaplan-Meier-estimate, HR, CI, p-value, Fig. 2
A3: Univariate	Varies	OS (?)	M1-M4, v3-v5, v8, v10, v11	HR, CI, p-value, Tab. 3[2]
A4: Multivariable M1 vs M4, M2 vs M4, and M3 vs M4	Varies but unknown	OS (?)	adjusted for v3-v5, v8, v10, v11	HR, CI, p-value, Tab. 4

[1] Tested for NRAS mutation only in patients with wtKRAS and wtBRAF.

[2] Only significant analyses shown in Table 3. What about others, e.g. v7: non-significant? No statement!

Additional: NRAS patients treated with anti-EGFR monoclonal antibodies	8	Median OS and PFS		See page 87
Statistical software packages used			No information given	

Martin et al (2015) Diagnostic criteria for the classification of cancer-associated weight loss. *J. Clin. Oncol.* **33**: 90-99, doi:10.1200/JCO.2014.56.1894

A. Patients, treatment, and variables

Patients: Cancer patients in supportive/palliative care or surgical or medical oncology, T: 12 institutions in 9 countries; V: Montpellier, France	
**T: 8737, V: 3075**	Patients assessed (data from 12 (training T) and 1 (validation V) data sets - Tab. 1)
**T: 577, V: 382**	Patients excluded (weight gain (T: n=515), age<18, carcinoma in situ, missing weight loss and BMI data, missing survival data)
**T: 8160, V: 2693**	Patients included for analysis
Treatment and follow-up: Routine clinical practice. Median-follow-up T 41.3 months, V 25.7 months	
Markers:	M1=BMI (cont.), M1(10)=BMI (10 cat: deciles), M1(5)=BMI (5 cat), M2=PWL (% weight loss, cont.), M2(11)=PWL (11 cat: deciles PWL + weight stable), M2(5)=PWL (5 cat), M3=BMI-adjusted WL grading system (5 cat), simplifying 25(m1(5) x m2(5)) combination by similarity in survival
Outcomes (events):	OS (T: 6294, V: unclear: 1713-1716, see Tab. 4 – 1713 deaths, but n only 2690)
Further variables:	v1=age (cont.), v1b=age (binary, cutpoint 65), v2=sex, v3=cancer site (‘diagnosis’), v4=cancer stage, v5=performance status, v6=health care setting, v7=weight*, v8=height*

*not used in any survival analysis, given in Tab. 2
Abbreviations: OS=overall survival, defined as time from WL and BMI assessment and death.

B. Statistical analysis of survival outcomes

Aim	n	Outcome (Events)	Variables considered	Results/ remarks
A1(T): Univariate	Varies due to missing	OS (varies)	M1, M2, v1, v2-v5	median OS (+CI), HR(+CI), p-value, Tab. 3
A2(T): Multivariable	? perhaps 7015 (see v5, Tab. 2)	OS (?)	M1, M2, v1, v2-v5	HR (+CI), p-value, Tab. 3
A2.5(T): Univariate weight loss over time frames	8137	OS (6279)	M2	p-values, data not shown. Results p.94
A3(V): Multivariable	? perhaps 2598 (see v4, Tab. 2)	OS (?)	M1, M2, v1, v2-v5	HR (+CI), p-value, Tab. 3
A4(T): To simplify BMI(10) and PWL(11) to BMI(5) and PWL(5)	8160 (M1), 8138* (M2)	OS (6294, 6279)	M1(10), M2(11)	Median OS, HR, indication of significance (p<.05) between deciles, Fig. 1, Text section ‘BMI Adjusted…’

A5(T): 25 (M1(5) x M2(5)) subgroups, to derive M3 - combining groups with similar HRs	8138*	OS (6279)	M1(5), M2(5)	Median OS (+CI), HR, 5x5 matrices, indication of significance (p<.05) between cells, Fig. 2 (+legend), Text p.95
A6(T): Separation by M3	8138*	OS (6279)	M3	c-statistic (+CI), Text p.95
A7(T): Effect of M3, multivariable	Unknown (8138 in M3, but missing)	OS (?)	M3, v1, v2-v5	HR (+CI), p-values, Text p. 95
A8(T): Effect of standard factors in subgroups defined by M3, univariate	Varies	OS (varies)	M3, v1b, v3-v6	For 'variables with adequate sample size', median OS (+CI), p-values (for trend?), Tab. 4
A9(T): Effect of standard factors in subgroups defined by M3, multivariable	Varies	OS (varies)	M3, v1, v1b, v3-v6	p-values (for trend?), Tab. 4 adjusted for v1-v5
A10(T): Effect of M3 in selected subgroups defined by v3	A: 947<br>B: 993	OS (A=804, B=455)	M3, v3	Kaplan-Meier estimates, p-values, 2 (highly selected?) subgroups presented, Fig. 3
A11(V): Separation by M3	2690	OS (1713)	M3	c-statistic (+CI), Text p.97
A12(V): Univariate	2690	OS (1713)	M3	p-value (for trend?), Tab. 4
A13(V): Multivariable	? perhaps 2598 (see v4, Tab. 2)	OS (?)	M3	p-value (for trend?), Tab. 4 adjusted for v1-v5
A14(T): As A2 – estimates in subgroup (v4=III or IV)	? perhaps 7284 (see v4, Tab. 3)	OS (perhaps 5953, see v4, Tab. 3)	M1, M2, v1, v2, v3, v5	HR (+CI), p-value, legend a bit misleading, Tab. A1
A15(T): As A7 – estimates in subgroup (v4=III or IV)	? perhaps 7284 (see v4, Tab. 3)	OS (perhaps 5953, see v4, Tab. 3)	M3, v1, v2, v3, v5	HR (+CI), p-value, legend a bit misleading, Tab. A2
Statistical software packages used:			SPSS 22.0	

*8137 in Tab. 3, 8138 in Fig. 1 and 2

Ostronoff et al (2015) Prognostic significance of NPM1 mutations in the absence of FLT3-internal tandem duplication in older patients with acute myeloid leukemia: a SWOG and UK National Cancer Research Institute/Medical Research Council report. *J. Clin. Oncol.* **33**: 1157–1164, doi:10.1200/JCO.2014.58.0571

A. Patients, treatment, and variables

Patients: Older patients with acute myeloid leukaemia (AML) data from four Southwest Oncology Group (SWOG, 1992-2009) and five UK National Cancer Research Institute/ Medical Research Council (MRC/NCRI, 1988-2010) trials	
**?**	Patients previously untreated, treatment with intensive chemotherapy, age ≥ 55 years
**?**	Patients excluded (acute promyelocytic leukemia)
**156** (SWOG)<br>**1258** (MRC/NCRI**)**	Patients included for analysis (SWOG: training population; MRC/NCRI: validation population; combined population for analyses A11-A13)
Treatment and follow-up: Intensive chemotherapy. Follow-up length not mentioned	
Markers:	M1=NPM1 (+/-), M2=FLT3-ITD (+/-), M3 (combination of M1 and M2)=M1+ and M2- vs. others
Outcomes (Events):	O1: Overall survival (OS, n=?)<br>O2: Relapse free survival[1] (RFS, n=?)<br>O2: 2-year OS (rates given, no numbers of events[2])<br>O3: 2-year RFS (rates given, no numbers of events)<br>O4: Therapy-related mortality (TRM, SWOG: n=0; NCRI: n=131)<br>O5: Complete remission (CR, SWOG: n=97; NCRI: n=903)<br>O6: 1-year relapse rate (SWOG: n=45; NCRI: n=378)
Further variables:	v1cont=age (cont.), v1cat=age (binary, cutpoint 65), v2=WBC count, v3=BM blast percentage, v4=sex, v5=ECOG PS (0-1, >1), v6=cytogenetics (unfavourable/other), v7=secondary AML[3], v8=platelet count, v9=IDH1/2, v10=DNMT3A

Abbreviations: NPM1=nucleophosmin, FLT3-ITDs=internal tandem duplications in FMS-related tyrosine kinase 3, WBC=white blood cell, BM=bone marrow, ECOG PS=Eastern Cooperative Oncology Group performance status

[1] As measured from the date of complete remission; thus, RFS only calculated for CR cases (n=97)
[2] Rates for 2-year OS and 2-year RFS are mislabelled as numbers of events in Tabs. 1, A1, A3 and A4 (probably a formatting error in the tables).
[3] Result of treatment of previous (other) cancer, diagnosis before start of treatment, only used in descriptive analyses

B. Statistical analysis of survival outcomes

Aim	n	Outcome (events)	Variables considered	Results/ remarks
Description, SWOG	156		v1cont, v2-v8, M1-M3, outcomes	Results, Tab. A1
A1(SWOG): Multivariable analysis	? (due to missing for v5 and v6)	OS (?), RFS (?)	M1, M2, v1cont, v2-v6, v8	HR (+CI), p-values, Results, Tab. A2
IDA1(SWOG): Relationship of v1cat with other variables	156		M1, M2, M3, v1cat, v2-v6, SWOG study#	2 x k tables, p-values, Tab. 1
A2(SWOG): Univariate, influence of v1cat on outcome	Varies	TRM (0), CR (97), 2-year OS (?), 2-year RFS (?) 1-year relapse (45)	v1cat	p-values, Tab. 1
A3(SWOG): Multivariable, in v1cat subgroups	?	OS (?), RFS (?)	M1, M2, v1cat, v2-v6, v8	HR (+CI), p-values, Tab. 2 As A1, but in subgroups
A4(SWOG): univariate	OS 156, RFS 97	OS (?), RFS (?)	M3, v1cat	Kaplan-Meier estimates, p-values, Figs. 1A, 1B, A1A, A1B
A5(SWOG): Multivariable, M3 in v1cat subgroups	?	OS (?)	M3, v1cat, v2-v6, v8	HR (+CI), p-values, Tab. 3
A6(SWOG): Multivariable, test for interaction between v1 and M3	156	-	M3, v1cat, other variables?	p-value, Results Mentions “multivariable”, but which variables included?
A7(SWOG): Univariate, in v1cat and SWOG study# subgroups to control for treatment	98	OS (?), RFS (?)	M3	Kaplan-Meier estimates, p-values, Figs. 1A, 1C, A1C
IDA2(SWOG): Relationship of v1cat with other variables, but in subgroup M3-positive	32		v1cat, v2-v7, v9, v10	2xk tables, p-values, Tab. 4. Results as for IDA1, but in M3-positive subgroup

A8(SWOG): Univariate, influence of v1cat on outcome, in subgroup M3-positive	32	TRM (0), CR (23), 2-year OS (?), 2-year RFS (?), 1-year relapse (?)	v1 cat	Kaplan-Meier estimates, p-values, Tab. 4, Figs 1D, A1D, A2. Results as for A2, but in M3-positive subgroup
IDA3(NCRI): Relationship of v1cat with other variables	1258		M1-M3, v1cat, v2-v6	2xk tables, p-values, Tab. A3, Results as for IDA1, but in validation population
A9(NCRI): Univariate, influence of v1cat on outcome	1258	TRM (131), CR (903), 2-year OS (?), 2-year RFS (?), 1-year relapse (374)	v1cat	p-values, Tab A3
IDA4(NCRI): Relationship of v1cat with other variables in subgroup M3-positive	209		M1-M3, v1cat, v2-v6	2xk tables, p-values, Tab. A4, Results as for IDA2, but in validation population
A10(NCRI): Univariate, as A8 in subgroup M3-positive	209	TRM (18), CR (178), 2-year OS (?), 2-year RFS (?), 1-year relapse (44)	v1cat	Tab A4, Kaplan-Meier estimates, Figs. 2A, 2B, A2
A11(combined): Prognostic significance of M3 in subgroup CN-AML*	743	OS (?)	M3, v1cat	Kaplan-Meier estimates, p-values, Figs. A3A, A3B, Results
A12(combined): Univariate, subgroup M3-positive patients in CN-AML	202	OS (?)	M3, v1cat	Kaplan-Meier estimate, p-value, Fig. A3C, Results
A13(combined): Multivariable, test for interaction between v1cont and v6	1414		M3, v1cat (probably, not explicitly stated), other variables?	p-value, Results
Statistical software packages used:	No information given			

*CN-AML = cytogenetically normal acute myeloid leukaemia, i.e. no clonal abnormalities in ≥20 metaphases analysed

Xing et al (2015) Association between BRAF V600E mutation and recurrence of papillary thyroid cancer. *J. Clin. Oncol.* **33**: 42–50, doi:10.1200/JCO.2014.56.8253

A. Patients, treatment, and variables

Patients: Consecutively-selected patients treated for papillary thyroid cancer (PTC) at 16 medical centers in 8 countries (USA, Italy, Poland, Japan, Australia, Spain, Czech Rep, South Korea), over differing time periods spanning 1978-2011.	
**?**	Patients assessed
**?**	Patients excluded
**2099**	Patients included for analysis, subgroups by v8 (v8-S1: CPTC, n=1448; v8-S2: FVPTC, n=431), v9 (v9-S1: Stage I, n=1273; v9-S2: Stage II, n=234) and v4 (v4-S1: tumor size ≤1.0cm, n=534)<br>Missing data not mentioned – appears to have been none
Treatment and follow-up: Total thyroidectomy and neck dissection in all patients, postoperative hormone suppression and radioiodine ablation (in all centers except the Japanese center). Median follow-up 36 months (IQR 14 to 75 months)	
Marker:	M = BRAF V600E mutation(positive/negative)
Outcome (events):	Recurrence free survival (RFS, events overall: n=338; in subgroups: v8-S1: n=247, v8-S2: n=43, v9-S1: n=119, v9-S2: n=32, v4-S1: n=57. Expressed as both a proportion of recurrences, and as rate of recurrence per 1000 person-years of follow-up
Further variables:	v1=age, v2=sex, v3=medical center, v4=tumor size, v5=extrathyroidal invasion, v6=lymph node metastasis, v7=multifocality, v8=PTC subtype, v9=tumor stage<br>Adjustment model 2: v1-v3; model 3: v1-v8

Abbreviations: CPTC=conventional PTC, FVPTC=follicular-variant PTC

B. Statistical analysis of survival outcomes

<table>
<tr><th colspan="2">Aim</th><th>n</th><th>Outcome (Events)</th><th>Variables considered</th><th>Results/ remarks</th></tr>
<tr><td colspan="2">C1: Check of proportional hazards assumption after initially fitting models A2 and A3</td><td></td><td></td><td></td><td>Led to stratification by medical center (v3) and revision of these analyses</td></tr>
<tr><td colspan="2">IDA1: Computation of rates of recurrence per 1000 person-years</td><td>Total and all subgroups</td><td></td><td></td><td>Displayed in Tables 2, 4 and A3</td></tr>
<tr><td rowspan="3">A1: Univariate unadjusted model 1</td><td>All</td><td>2099</td><td>RFS (338)</td><td rowspan="3">M</td><td rowspan="3">Poisson regression p-values and CI; Cox regression HR, CI: Tab. 2; Kaplan-Meier estimates of recurrence-free survival, p-values: Fig. 1</td></tr>
<tr><td>v8-S1</td><td>1448</td><td>RFS (247)</td></tr>
<tr><td>v8-S2</td><td>431</td><td>RFS (43)</td></tr>
</table>

A2: Multivariable model 2	All	2099	RFS (338)	M, v1-v3	p-values, HR, CI, Tab. 2
	v8-S1	1448	RFS (247)		
	v8-S2	431	RFS (43)		
A3: Multivariable model 3	All	2099	RFS (338)	M, v1-v8	p-values, HR, CI, Tab. 2
	v8-S1	1448	RFS (247)		
	v8-S2	431	RFS (43)		
A4: Sensitivity analysis, excluding patients with <3 year follow-up, no recurrence		?	RFS ?	M	Results p.44 Text. Data not shown
A5: Interaction of M with conventional risk factors, univariate		2099	RFS (338)	M, v1 (dichotomized), v5, v6	Kaplan-Meier estimates, p-values, Fig. 2, Synergy Index, CI, Tab. 3
A6: Low risk patients, unadjusted model 1	v9-S1	1273	RFS (119)	M	Poisson regression p-values and CI; Cox regression HR, CI: Tab. 4
	v9-S2	234	RFS (32)		
	v4-S1	534	RFS (57)		
A7: Low risk patients, multivariable model 2	v9-S1	1273	RFS (119)	M, v1-v3	p-values, HR, CI, Tab. 4
	v9-S2	234	RFS (32)		
	v4-S1	534	RFS (57)		
A8: Low risk patients, multivariable model 3	v9-S1	1273	RFS (119)	M, v1-v8	p-values, HR, CI, Tab. 4
	v9-S2	234	RFS (32)		
	v4-S1	534	RFS (57)		
A9: Univariate model in v4 subgroups		Varies by subgroup	RFS (Varies)	M	p-values, HR, CI, Tab. A2
A10: Univariate model for 35 subgroups by v1, v2 and v8		Varies by subgroup	RFS (Varies)	M	HR, CI, Tab. A4
Statistical software packages used:			SAS v.9.3		
